# Histologically resolved multiomics enables precise molecular profiling of human intratumor heterogeneity

**DOI:** 10.1371/journal.pbio.3001699

**Published:** 2022-07-01

**Authors:** Tao Chen, Chen Cao, Jianyun Zhang, Aaron Streets, Tiejun Li, Yanyi Huang

**Affiliations:** 1 Biomedical Pioneering Innovation Center (BIOPIC), School of Life Sciences, Peking University, Beijing, China; 2 College of Engineering, Peking University, Beijing, China; 3 Department of Oral Pathology, Peking University School and Hospital of Stomatology & National Center of Stomatology & National Clinical Research Center for Oral Diseases & National Engineering Research Center of Oral Biomaterials and Digital Medical Devices, Beijing, China; 4 Beijing Key Laboratory of Digital Stomatology; 5 Research Unit of Precision Pathologic Diagnosis in Tumors of the Oral and Maxillofacial Regions, Chinese Academy of Medical Sciences (2019RU034), Beijing, China; 6 Peking-Tsinghua Center for Life Sciences, Peking University, Beijing, China; 7 Beijing Advanced Innovation Center for Genomics (ICG), Peking University, Beijing, China; 8 College of Chemistry and Molecular Engineering, Peking University, Beijing, China; 9 Institute for Cell Analysis, Shenzhen Bay Laboratory, Guangdong, China; Centro de Investigacion y de Estudios Avanzados del Instituto Politecnico Nacional, MEXICO

## Abstract

Both the composition of cell types and their spatial distribution in a tissue play a critical role in cellular function, organ development, and disease progression. For example, intratumor heterogeneity and the distribution of transcriptional and genetic events in single cells drive the genesis and development of cancer. However, it can be challenging to fully characterize the molecular profile of cells in a tissue with high spatial resolution because microscopy has limited ability to extract comprehensive genomic information, and the spatial resolution of genomic techniques tends to be limited by dissection. There is a growing need for tools that can be used to explore the relationship between histological features, gene expression patterns, and spatially correlated genomic alterations in healthy and diseased tissue samples. Here, we present a technique that combines label-free histology with spatially resolved multiomics in unfixed and unstained tissue sections. This approach leverages stimulated Raman scattering microscopy to provide chemical contrast that reveals histological tissue architecture, allowing for high-resolution in situ laser microdissection of regions of interests. These microtissue samples are then processed for DNA and RNA sequencing to identify unique genetic profiles that correspond to distinct anatomical regions. We demonstrate the capabilities of this technique by mapping gene expression and copy number alterations to histologically defined regions in human oral squamous cell carcinoma (OSCC). Our approach provides complementary insights in tumorigenesis and offers an integrative tool for macroscale cancer tissues with spatial multiomics assessments.

## Abstract 2

The composition and spatial distribution of cells in the tumor microenvironment play a critical role in both disease progression and diagnosis. This study presents a new platform that combines label-free histology with genomic analysis, allowing spatially resolved characterization of genomic and transcriptomic heterogeneity in human oral squamous cell carcinoma.

## Introduction

Recent technological developments for low-sample input genomic analysis have led to high-resolution characterization of cellular heterogeneity in tissues and organs. Specifically whole-genome and whole-transcriptome amplification have enabled comprehensive molecular profiling of single cells [1,2]. Most techniques for single-cell genomic analysis require the dissociation of millimeter-scale biological samples into a single-cell suspension, before the extraction of genomic material, limiting the spatial resolution of these techniques to the size of the dissected tissue sections [3,4]. While high-throughput single-cell RNA sequencing has been a powerful tool for classifying cell types and revealing cell states, cellular state and function are strongly influenced by the cell’s microenvironment, including chemical and physical context as well as the composition of neighboring cells [5,6]. Therefore, comprehensive cellular characterization of biological systems would ideally leverage the combination of genome-wide molecular analysis and high spatial resolution. Laser capture microdissection (LCM) [7–9] enables the recovery of submillimeter regions of interest (ROIs) from tissue, allowing for microscopy to guide the selection of cells based on the morphology of the microenvironment. In this way, LCM links histology with molecular measurements, including genome and transcriptome-wide profiling with high-throughput sequencing. However, LCM typically requires fixation and staining to generate histological contrast, and these steps can perturb or even degrade the nucleic acid composition of cells [10,11]. Furthermore, the contrast used to guide dissection in these applications is limited by the chosen staining method [12].

**Fig 1:**
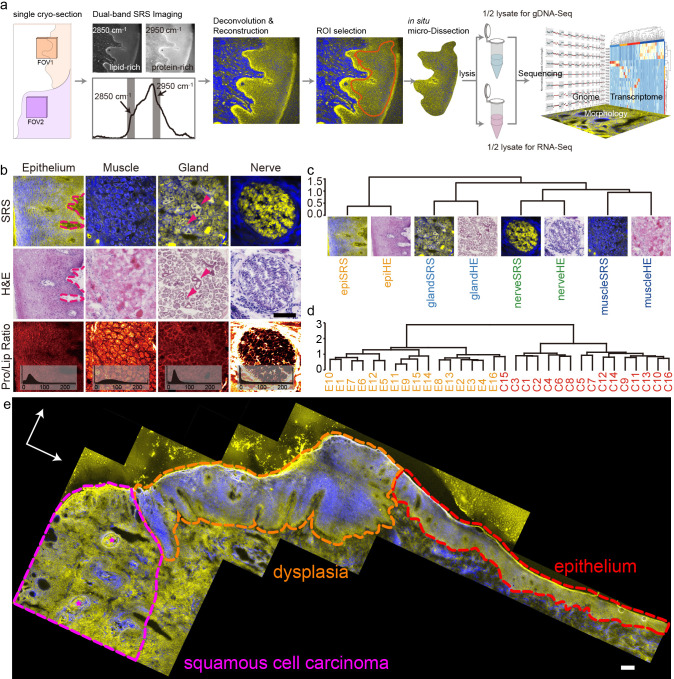
Principle of SMD-seq and evaluation of SRS imaging. (a) The pipeline of SMD-seq, inset of FOV2 represents the complexity around single cancer nest. (b) The comparison between SRS and HE images of different tissues, with the PLR images of SRS. (c) Unsupervised clustering of HOG features of SRS and HE images from different tissue types. (d) Unsupervised clustering of SRS images of epithelium and cancer. (e) Stitched SRS image of a whole sample, showing revealed morphologies including cancer, dysplasia, and epithelium. The magenta stars indicate the keratin pearls. Scale bars are 200 μm. HE, hematoxylin–eosin; HOG, histogram of orientation gradient; PLR, protein-to-lipid ratio; SMD-seq, SRS microdissection and sequencing; SRS, stimulated Raman scattering.

Here, we present an integrative approach for mapping molecular and cellular heterogeneity in tissue samples that leverages stimulated Raman scattering (SRS) microscopy [13] to generate histological images of fresh frozen tissue slices followed by in situ microdissection and multiomic sequencing analyses. This technique, called SRS microdissection and sequencing (SMD-seq), images tissue sections without staining, and instead provides contrast based on the intrinsic chemical composition of the biological specimen. SRS generates signal from molecular vibrations that are specific to chemical bond composition. Imaging a biological sample at multiple Raman frequencies can therefore provide composite images with morphological contrast that corresponds to chemical variation [14–17]. After label-free imaging of fresh or frozen tissue slices, ROIs are dissected directly with the SRS excitation laser, and immediately processed enabling efficient recovery of RNA and DNA from small numbers of cells within an ROI (Fig 1A, Materials and methods). The combination of quantitative chemical imaging and high-resolution genomic analysis facilitates multimodal characterization of the cellular heterogeneity in complex tissues.

The ability to map molecular and cellular heterogeneity is particularly important in cancer. Heterogeneity in cancer is expressed in many facets, including the evolving genetic changes in distinct subpopulations [18,19], and the progressive abnormalities in morphology of the cells, typically identified by histopathology [20]. Molecular and phenotypic aberrant variations are not only common between tumors of different tissue and cell types, but also within a tumor derived from the same patient or even from the same tissue [21]. The clonal diversity during tumorigenesis, in the context of histology and genetic divergence, influences each individual tumor’s diagnosis and response to treatment [22]. It is therefore important to characterize the tumor heterogeneity in a comprehensive and precise way.

In this study, we demonstrate the utility of SMD-seq by spatially mapping genomic and transcriptomic profiles in human oral squamous cell carcinoma (OSCC). OSCC is a major subtype of head and neck squamous cell carcinoma (HNSCC). Its aetiological and biological heterogeneity are influenced by distinct risk factors [23,24], which further lead to high morbidity and relatively low overall 5-year survival rate. OSCC is highly invasive, leading to large variation in cellular phenotypes over small length scales [25,26]. OSCC cancer nests, for example, measuring as small as 100 μm in diameter, are often interlaced with normal tissues composed of noncancerous cells, and it is challenging to dissect this heterogeneity with existing methods [25]. Additionally, mutation patterns in OSCC have been shown to differ from other subsites of HNSCC due to its specific anatomical location and tissue characteristics [27]. We used SRS histology to identify progressive abnormalities in the morphology of OSCC cancer cells, and in situ laser microdissection followed by DNA and RNA sequencing identified unique molecular profiles that correspond to distinct anatomical regions of tissue samples from multiple individuals. Transcriptomic analysis was used to identify gene expression profiles that corresponded to specific tissue types and cancer cells, which corroborated histological analysis. Furthermore, transcriptomic and genomic analysis revealed gene fusion events and copy number variations that delineated cancer progression within tissue sections. This combination of imaging and multiomic analysis demonstrates how SMD-seq can provide a powerful tool to accurately investigate complex inter- and intratumor heterogeneity and has the potential to be scalable to any architecturally complex tissue.

## Results

### Label-free SRS microscopy accurately revealed histological features of OSCC

The recent and rapid development of SRS-based histology [14–17] has proven this technique to be a powerful supplement to traditional histological staining for many tissues. We first examined the ability of SRS imaging to reconstruct histological features in unstained cryo-sections of OSCC that corroborated information obtained with hematoxylin–eosin (HE) staining of OSCC sections. We generated 2-color SRS composite images of 30 μm cryo-sections biopsied from various tissues that provide contrast between lipid-rich and protein-rich structures by exciting CH_2_ symmetric vibration and CH_3_ vibrations, respectively (Materials and methods, S1 Fig). To validate the efficacy of SRS-based histology for OSCC, we then compared these reconstructed images to adjacent 5 μm cryo-sections that were sampled from the same biopsies and conventionally stained by HE. The lipid–protein contrast in the SRS images recovered the characteristic morphological features of each tissue, including the variation of cell shape and base membrane in the epithelium, the epithelial cells forming the duct wall in the gland tissue [28] and the nerve bundles (Fig 1B). In addition to recapitulating morphological information, SRS images provide information about the chemical composition of the tissue samples. For example, both the muscles and the perineurium, a collagen-rich structure [29,30], around nerve revealed high protein content in SRS images (Fig 1B). Variability in the chemical composition of ROIs can be further evaluated by the histogram of the protein-to-lipid ratio (PLR) from different tissue types, reflecting a distinct molecular signature of the cells within the tissue. In order to confirm that the histopathological utility of SRS was not limited to OSCC, we also identified characteristic features of 2 other oral cavity diseases, the Warthin’s tumor and the mucoepidermoid carcinoma (S2 Fig). These results demonstrate the generalizability of SRS imaging for oral cavity histology.

We then quantified the similarity between morphological information retained in SRS and HE images using the histogram of orientation gradient (HOG) [31]. The HOG describes the texture of an image based on intensity variation, which primarily reflects cellular packing in our images. Unsupervised clustering of the HOG from all 8 images presented in Fig 1B, successfully clustered images by tissue type, regardless of imaging modality (Fig 1C). This analysis indicates that SRS images recovered similar cellular organization patterns as HE images. In addition to distinguishing different types of tissue, the ability to distinguish cancerous epithelium from normal epithelium has potential clinical implications. The cellular organization patterns between these tissue states are distinct and can be decoded by HOG features. Unsupervised clustering of the HOG features of 32 SRS images, including 16 cancerous and 16 normal epithelia (S3 Fig), stratified tissue images into 2 groups, accurately reflecting disease state with only 1 misidentification (Fig 1D). Furthermore, the correlation matrix of all 32 samples’ HOG features revealed a weaker correlation among cancers than epithelia (S4 Fig), implying greater variation in cellular organization patterns between individual cancer nests. This heterogeneity could be related to the distinct microenvironment of the cancer nests. For example, the cancer nests in P4 invaded heavily into muscle tissue, while in other patient samples the cancer nests were typically located in connective tissue (S5 Fig). We examined the morphology restoration at different scales on stitched image of a whole cryo-section (9 × 4 mm^2^). Comparing with corresponding HE images, SRS revealed identical features at all scales. Selected ROIs from SRS images can be localized in HE images through correlation of their respective HOG (Figs 1E and S6). The key morphological features were identified in the large-scale SRS image depicted in Fig 1E, including normal epithelium, dysplasia, cancer, and those keratin pearls correlated with OSCC differentiation.

### SRS guided microdissection enabled high-quality mRNA and DNA sequencing of cancer nests

**Fig 2:**
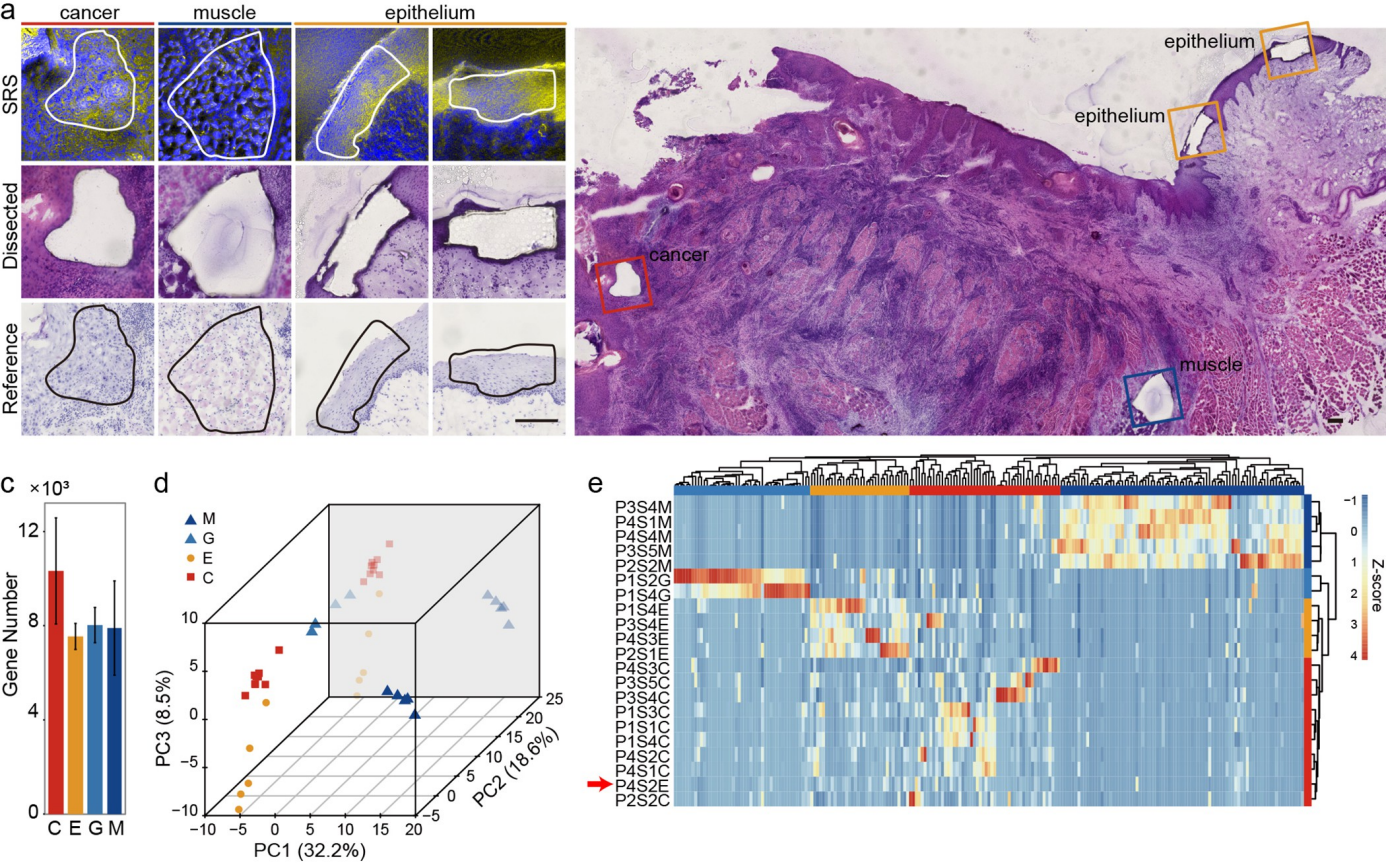
SRS image guided microdissection and evaluation of mRNA recovery. (a) Comparison between SRS images, HE-stained and unstained microtissue section. (b) Epithelium, muscle, and cancer from the same section. (c) Statistics of gene number per sample. (d) PCA plot of all expressed genes. (e) Unsupervised clustering of 217 differently expressed genes from microdissected samples. Scale bars are 200 μm. Source data for panels c–e in S2 Data Sheet 2.1–2.3. HE, hematoxylin–eosin; PCA, principal component analysis; SRS, stimulated Raman scattering.

In the SMD-seq workflow, after an ROI is identified with SRS histology, that region is immediately excised and recovered from the bulk tissue section for downstream genomic analysis. This is achieved by increasing the power of the pump line of the SRS excitation source and scanning the focal spot around a user-defined ROI boundary (Materials and methods). To validate the accuracy of ROI dissection, we performed HE staining on the remaining tissue section after dissection, as well as adjacent tissue sections (Fig 2A). Because microdissection is performed with the scanning laser, the ROI is defined with high resolution (S7A Fig) and can be recovered immediately after imaging. Depending on the ROI size and tissue type, the dissected sample typically contained about 230 cells on average (S7B Fig). SRS imaging and dissection can be performed in rapid succession, allowing the dissection of multiple ROIs from a single tissue section (Fig 2B), therefore when profiling cancer regions, all the cancer microtissues were paired with a normal epithelial microtissue from the same tissue section to control for intersample variability.

After recovery, each microsample was lysed and divided into 2 equal aliquots for parallel DNA and mRNA extraction. One of the primary advantages of SMD-seq is the efficient recovery of high-quality mRNA, which is made possible because SRS histology avoids fixation, staining, or any other chemical perturbations that may degrade RNA or inhibit recovery. Quantification of housekeeping genes showed that unstained cryo-sections can preserve over 20-fold more mRNA than HE-stained sections (S8 Fig). Another advantage of SMD-seq is that the implementation of whole-genome and whole-transcriptome amplification enables multiomic analysis from a single ROI. Finally, SRS histology and LCM enables molecular profiling on subregions of a tissue section so that cellular heterogeneity can be reduced and ensemble measurement averages over a smaller ROI. Comparing with HNSCC samples collected for the Cancer Genome Atlas, (TCGA), the microsamples measured with SMD-seq presented significantly lower cell-type diversity (*P* value = 0.0098, S7C–S7E Fig, Materials and methods). The roughly 100-μm resolution ROI subselection in SMD-seq allows for profiling of intrasection heterogeneity; however, the lack of single-cell resolution within the microsamples means that some heterogeneity is still obscured.

We applied SMD-seq to 13 cryo-sections from 4 patients (3 males, 1 female) who suffered various stages of OSCC at different ages (Materials and methods). We collected 28 in situ microdissection samples for sequencing, and 27 samples passed the quality control based on mRNA recovery, for further analysis (S1 to S3 Tables, Materials and methods). These microsamples included 12 normal (5 muscle, 2 gland, and 5 epithelium) and 9 cancer samples for RNA-seq and 13 normal (muscle, *n* = 5; gland, *n* = 2; and epithelium, *n* = 6) and 8 cancer samples for DNA-seq. RNA-seq recovered around 9,000 genes per microsample on average (FPKM > 0.1, Fig 2C) and highly correlated expression among samples of the same tissue, indicating technical reproducibility (S9 Fig).

**Fig 3:**
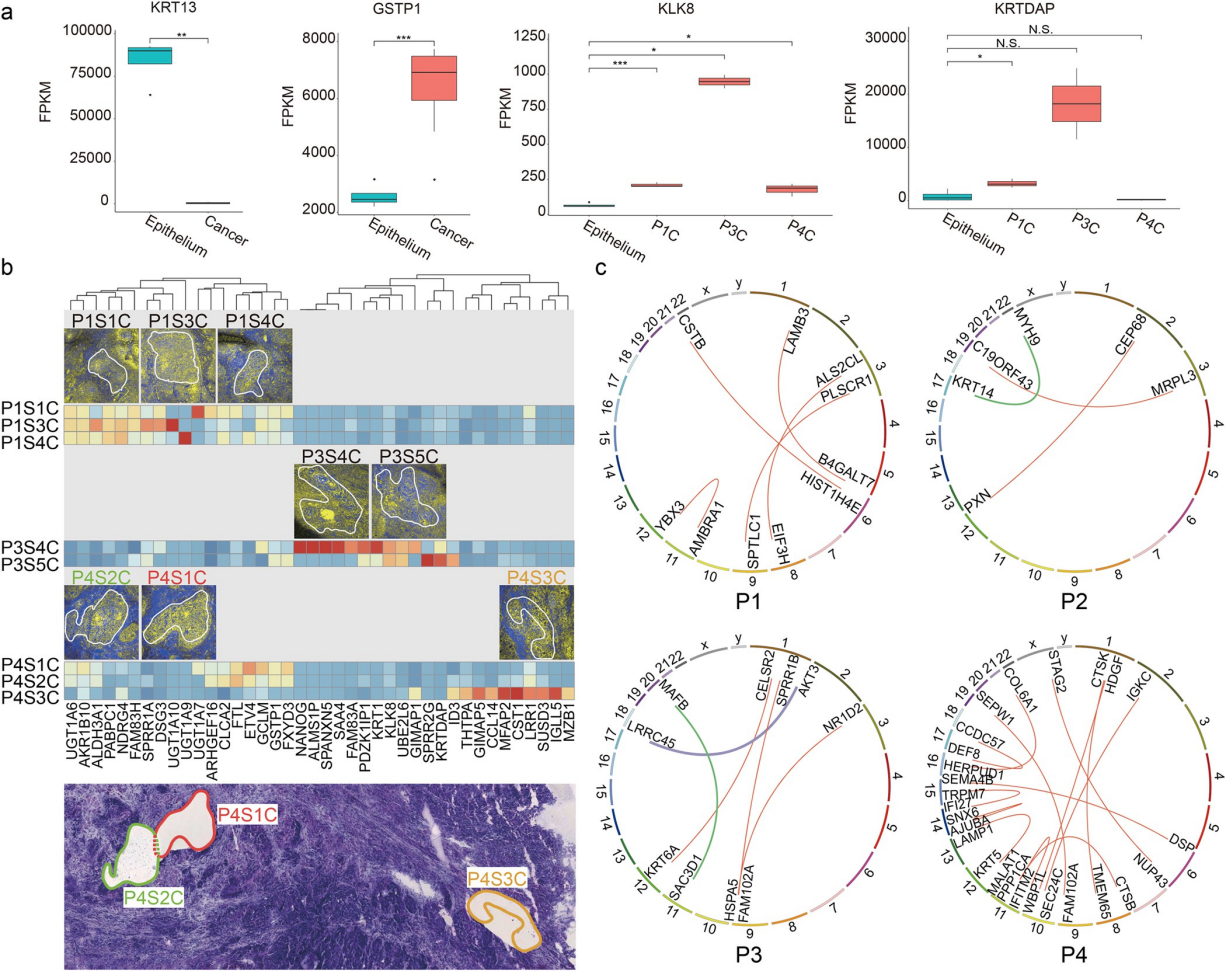
Heterogeneity of gene expression. (a) Expression levels of genes in cancer samples among different patients, in comparison with epithelium. (b) The interpatient and intrapatient cancer heterogeneity. The bottom figure is a projection of 3 slides, showing the relative positions of different cancer nests. (c) Interpatient heterogeneity of gene fusion events. Source data for panel a in S2 Data Sheet 2.4.

Principal component analysis (PCA) separated microsamples into 4 groups of similar gene expression profiles that recapitulated the known tissue types (Fig 2D). Unsupervised hierarchical clustering of microsamples using the top 217 differently expressed genes (Materials and methods) grouped these clusters as distinct tissue types, 3 of which were then annotated using a panel of known marker genes for epithelium, gland, and muscle in agreement with the corresponding tissue types identified by SRS histology (Figs 2D and S10). The fourth group, which was consistently identified as cancer by SRS images, contained highly expressed OSCC marker genes including GSTP1, AKR1B10, FTH1, and FTL (S11 Fig). GSTP1 expression is known to be associated with high malignancy and poor survival rate [32,33]. Expression levels of GSTP1 were further validated with immunofluorescence staining, confirming the confinement of GSTP1 expression to cancer nests (S12 Fig). While the epithelial to mesenchymal transition (EMTs) related gene, such as KRT13 (Fig 3A) [34], was expressed at lower levels, indicating an enhanced migratory capacity and invasiveness [35]. While these OSCC samples originated from the epithelium, they revealed a distinct expression profile compared to the healthy epithelium samples. Unexpectedly, P4S2E, which was identified as epithelium by SRS imaging, clustered with the cancer samples based on gene expression profiles (Fig 2E, red arrow). PCA also indicated that P4S2E was more similar to the cancer samples than the epithelium samples (Fig 2D). This observation indicates that microsample P4S2E may have come from a region of the biopsy in which these epithelial cells were developing into carcinoma. This level of molecular characterization is enabled by joint histological profiling with SRS and gene expression analysis.

### SMD-seq profiles molecular heterogeneity in OSCC

SMD-seq provides the ability to compare the genome and transcriptome of specific tissue types and disease states within a single tissue section, allowing for a more precise deconvolution of genetic profiles and gene expression profiles than traditional bulk assays. While healthy tissue showed consistent gene expression across samples, OSCC microsamples revealed large patient-to-patient variability in gene expression. KLK8 and KRTDAP only exhibited high expression in patient P3 (Fig 3A). KLK8 is implicated in malignant progression of OSCC [36], and KRTDAP strongly correlate with the differentiation and maintenance of stratified epithelium. Correspondingly, “keratin pearls,” structures indicating high-degree differentiation were present in SRS images of P3 (S13 Fig) [37]. In addition to variability between patients, we found significant variability in gene expression between samples identified as cancer nests from the same patient. Specifically, in P4, the adjacent P4S1C and P4S2C microsamples exhibited similar gene expression; however, P4S3C, which was recovered from a more distant location to previous 2 (S14 Fig), revealed a distinct gene expression profile including expression of CST1, which is related to promoted cell proliferation (Fig 3B). The intratumor molecular variation may indicate a branched evolution of tumorigenesis that could provide insight for tumor diagnosis and treatment.

In order to characterize the cellular heterogeneity within microsamples, we aimed to deconvolve their composition using a published single-cell dataset that profiled transcriptomes of 6,000 single cells from 18 HNSCC patients [38]. We first embedded the RNA-seq data from our tumor microsamples into 20 principal components that capture gene expression variation in the single-cell data from Puram and colleagues (S15A Fig). Most of the cancer microsamples colocalized with the malignant epithelium cluster in the single-cell data, indicating similar gene expression profiles between our sections and individual malignant cells. However, 1 sample colocalized with the fibroblast and endothelial cell clusters. We investigated the cellular composition of these samples, which collectively contribute to their gene expression profile, by identifying the top 1,000 nearest neighbor cells from the Puram data. Then, for each sample, we calculated the percent of these top 1,000 cells that fell into each of the annotated cell clusters (S15B Fig). Five of our microsamples were composed primarily of malignant cells; however, the remaining 3 showed a more diverse composition including tumor resident fibroblasts and T cells. Interestingly, most of the nearest neighbor cells to sample P4S3C were indeed fibroblasts and endothelial cells.

Displacement and recombination of genes, especially oncogenes, has been recognized to drive neoplasia [39] and has become the focus of numerous cancer studies as potential therapeutic targets [40–42]. We then exploited de novo identification of gene fusion events through sequencing these samples. Early fusion events are usually sporadic and hence hard to identify with bulk sequencing. Furthermore, DNA sequencing of small samples with low input heavily relies on whole-genome amplification, which is prone to chimera formation and causes false identification of fusion events. We used RNA-seq of microsamples for de novo identification of gene fusion transcripts to investigate gene fusion events between patients and within tumor samples. Some fusion-related genes were shared between microsamples from separate patients, including KRT6A, which was shared between P1S2C and P3S5C, and FAM102A, which was shared between P1S3C, P3S5C, and P4S2C. Some samples, however, contained unique gene fusion patterns (Fig 3C, S1 Data 1.3 to 1.8). Most of these fusions might be passenger events that came along with cancer development and thus their actual consequences remain unknown. Among those, we found several sets that are consistent with previous observations, including 1 recorded in TCGA (MYH9 and KRT14, from P2) and 2 involving oncogenes AKT3 (fused with LRRC45, from P3) and MAFB (fused with SAC3D1, from P3) (S16 Fig, S2 Table, S3 Data). Sanger sequencing on amplified fragments harboring joint junction of fused genes confirmed these fusions between MYH9 (5′ fusion partner: exon 20) and KRT14 (3′ fusion partner: exon 8), AKT3 (5′ fusion partner: UTR), and LRRC45 (3′ fusion partner: intron), implying the capability of SMD-seq in recurrent fusion event discovery. Fusion events also revealed substantial intrasample heterogeneity (S17 Fig). For example, more fused genes were found in P4S3C, than in P4S1C and P4S2C which were dissected from distant regions of the tissue section, indicating spatially dependent genome instability during tumorigenesis. Moreover, we also observed the existence of an oncogene-involved fusion (RAB3D and MTMR14, verified by Sanger sequencing, S3 Data) in sample P4S2E (S18 Fig), which was identified as healthy epithelium by HE and SRS analyses, again indicating the possibility that this microsample was progressing through the early stages of tumorigenesis.

**Fig 4:**
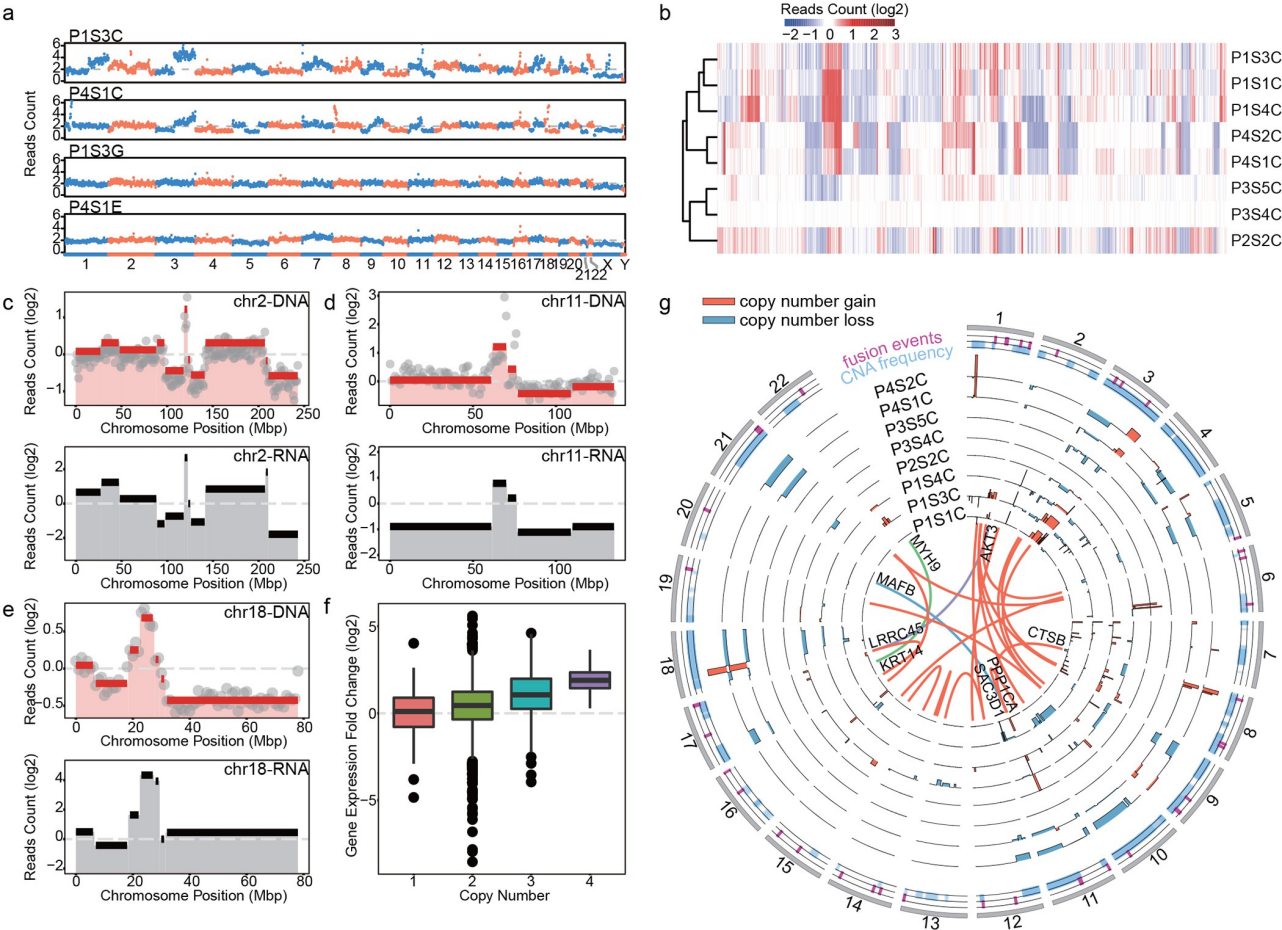
Genomic and transcriptomic analysis of laser dissected tissue samples. (a) Raw reads count across the whole genome of 2 paired cancer-normal tissues dissected from the same slice. Chromosome numbers were labeled at the bottom. (b) Unsupervised clustering of normalized reads counts of all the dissected cancer samples. (c, d) Top panels demonstrated the normalized read count (gray dots) and copy numbers (red lines) identified by CBS algorithm in chromosome 2 and 11 of sample P1S3C and P1S1C, respectively. Mean expression levels within the same segment were shown in black lines in the bottom panel. (e) Averaged read counts of all the cancer samples (top) and their corresponding gene expression values (bottom) of chromosome 18. Genome and transcriptome variations of other chromosomes and samples were shown in S16, S18, and S19 Figs. (f) Averaged gene expression fold changes were computed per 1 M bin across the whole genome and plotted against copy numbers. (g) The distribution of CNA and fusion genes across the genome of all the cancer samples. Orange and blue bars indicated the copy number gains and loss, respectively. Orange lines in the inner circle indicated fusion genes with at least 10 span pair reads, and the green and purple lines represented oncogenes involved gene fusions. The position of fusion events (magenta) and CNAs (light blue) of all the cancer samples were shown in the 2 outmost circles. Source data for panel b–g in S2 Data Sheet 2.5–2.20. CNA, copy number alteration.

In parallel to RNA-seq, DNA-seq was performed using half of the lysate from microsamples, enabling the analysis of heterogeneity at the whole-genome level. Copy number alteration (CNA) analysis demonstrated unique patterns of CNAs between cancer and normal samples, and between patients (Figs 4A and S19), indicating the high complexity in OSCC including significant genetic mosaicism and genetic heterogeneity. We subsampled reads to characterize library complexity and read depth, demonstrating the 0.1× was sufficient for accurate and reproducible CNA (S20 Fig). A few commonly shared large-size ploidy shifts, such as the losses of 3p and 8p and the gains of 3q and 8q, which are shared in other squamous cell carcinomas [43,44], were also observed in our OSCC samples. This analysis also revealed patient-specific CNAs, for example, chromosome 6 showed high instability in P2’s cancer sample but this instability was not present in others (S21 and S22 Figs). Unsupervised clustering of the samples based on CNAs further demonstrated the unique CNA patterns between patients (Figs 4B and S21) [45]. Microsamples dissected from different locations within a patient sample displayed subtle discrepancies in copy number profiles (Fig 4B). The CNA pattern of chromosome 1 in P1S1C and P1S3C were different from that of P1S4C, with an obvious gain of 1q in the first 2 ROIs (S22 Fig).

As both genomes and transcriptomes were sequenced from each cancer nest, we were able to perform joint analysis of genomic and transcriptomic variation. The mean expression level of genes within each genomic segment [46] was compared with the copy number in the same regions (Figs 4C–4E, S22, and S23) and summarized across the whole genome (Figs 4F and S24). The average gene expression levels showed positive correlation with the copy number within the same segment (Fig 4F). Among all the OSCC samples, 8 regions of recurrent copy number gain and 5 regions of recurrent copy number loss were identified (q < 0.25, S25 Fig, S1 Data 1.9 to 1.10) [43]. Among these (copy number loss/gain or regions), 11q13.3, 8q24.3, 11p15.4, and 11q24.2 colocalized with differently expressed genes in cancer samples. GSTP1 located within the recurrent focal amplification of 11q13.3 [44,47], which implied that the high expression level of GSTP1 may be the result of increased copy number. FAM83H was also colocalized with a focal amplification region, 8q24.3, and it specifically expressed at higher levels in patient P1. TP53AIP1 and PKP3 both expressed at a lower level in all the patients and located in the regions of recurrent copy number loss 11q24.2 and 11p15.4, respectively. The expression level of GSTP1, FAM83H, TP53AIP1, and PKP3 were all reported to be involved in the development of cancer or affecting patients’ survival rate [44,48]. We also analyzed the global correlation between gene fusion events and copy number variations and found a high degree of overlap between them (Fig 4G). Fusion genes CTSB and PPP1CA colocalized with focal amplification regions 8p23.1 and 11q13.3, separately. CTSB was proved to be related to cancer progression and metastasis [49,50], and PPP1CA was reported to contribute to ras/p53-induced senescence [51]. Of the 24 pairs of detected fused genes, 17 pairs (approximately 71%) had at least 1 gene intersected with a focal CNA (S1 Data 1.11). The parallel observation of genomic rearrangement and gene expression fold change may illustrate that the instability of the cancer genome led to gene fusion events that were more likely to occur within amplification and deletion regions (Fig 4G) [52].

## Discussion

SMD-seq takes advantage of SRS microscopy, which can capture chemical information without staining, and low-input sequencing to give high-quality genome and gene expression data from well-defined histological regions. This technique enabled dissection of cancer heterogeneity across multiple measurement modalities, including morphology, genome alteration, gene expression, and gene fusion.

Obtaining quantitative molecular information about cell populations in their native environment is challenging with traditional microscopy or genomic analysis alone. The major challenge hindering the progress of these studies is technical: although sequencing analyses, especially single-cell sequencing enables the systematic identification of cell populations in a cancer tissue, most of such studies start with a dissociated cell suspension [53], which retains no spatial information from the sample of origin. On the other hand, current methods in surgical pathology lack the capability to efficiently isolate specific cell populations in complex tissues/tumors, which can confound molecular results. Spatially resolved transcriptomic profiling or “spatial transcriptomics” refers to a rapidly growing suite of tools that directly address the challenge of high-resolution spatial mapping of molecular heterogeneity [54,55]. These methods fall into 3 broad categories: (1) single-molecule FISH-based methods, which include seqFISH [56] and MERFISH [57]; (2) spatial barcoding and sequencing-based methods that include the 10× Visium platform based on Spatial Transcriptomics [58], DBiT-seq [59], and Slide-seq [60]; and (3) LCM-based approaches that includes our SMD-seq method. Moses and Pachter recently reviewed the spatial transcriptomics literature and created a comprehensive database that catalogues hundreds of methods and applications, annotating their unique advantages and disadvantages [55]. smFISH-based methods tend to have the highest spatial resolution, typically limited by diffraction. Spatial barcoding and sequencing methods are powerful for transcriptome-wide profiling of large tissue sections but with reduced spatial resolution compared to smFISH methods. Both of these types of methods typically rely on a combination of fixation and staining to combine transcriptomic analysis with histological features. SMD-seq provides a complimentary approach to these powerful systems, enabling multiomic molecular analysis of targeted ROIs without fixation or staining. S4 Table compares the unique advantages of a representative subset of spatial transcriptomics methods; reference [55] provides a much more comprehensive overview.

The morphological information recovered by SMD-seq is reconstructed from chemical contrast, unlike gene expression in other spatial transcriptomic approaches [54,55]. Therefore, recovery of histological features does not rely on data reconstruction through gene expression patterns or spatial barcoding. Such independence between morphology and gene expression can lead to previously unobserved characteristics of tumorigenesis. The discordance between the morphology and genomic profile of microsample P4S2E served an example. The sample was identified as normal epithelium by SRS histology, HE staining of an adjacent tissue section. However, the gene expression profile of P4S2E clustered more closely with the cancer samples (Fig 2G), and both the genetic and transcriptomic features of this sample reflected a cancer-like pattern. Furthermore, genes previously reported to be significantly mutated in OSCC, such as FAT1, PPP2R1A, PTEN, HRAS, and CREBBP28, are also found in P4S2E (S26 Fig). This inconsistency between imaging and sequencing could imply that tumorigenesis signature reflected by gene expression profiles may arise before of morphological characteristics, such observation would be difficult to be captured and confirmed by histology reconstructed through expression patterns.

Although SMD-seq can preserve morphology and sequence information with high quality, there are additional limitations. First, SRS imaging in SMD-seq can identify local tissue features; however, there is an intrinsic trade-off between the field of view and resolution of the SRS histology images. In this study, the lower numerical aperture of the objective lens compromised the sensitivity for minor change in subcellular structures, which hindered in depth image analysis. Increasing pixel density of image may reduce the noise level with higher spatial sampling rate (S27 Fig), but prolonged exposure times lead to a higher chance of sample ablation during imaging (S27 Fig) and bring higher risk of the RNA degradation during imaging. Another limitation of SMD-seq is the accessibility of SRS microscopy, which requires specialized light sources and sophisticated optical configurations making the technique difficult to implement in general biology and clinical labs. The integration of SRS microscopy into turn-key system should in principle facilitate adoption of SMD-seq in the clinics [61].

In summary, we have shown that SMD-seq can readily detect spatially dependent transcriptional variation and chromosomal alteration in unfixed tissue sections, and it can discover the subtle and rare genetic alteration events, such as gene fusions, copy number changes, and differentially expressed transcriptome, with high sensitivity and accuracy, underlying different histological features. In this study, we performed bulk genomic analysis on the dissected microsamples, allowing parallel DNA and RNA sequencing on the same ROI. This small-bulk pool-and-split approach masks the single-cell-level heterogeneity within the sample and blurs the relationship between the genome and transcriptome in single cells. SMD-seq could be extended to single-cell analysis with careful dissociation of microsamples. While they could limit the ability of multiomic characterization, such a development would provide higher resolution molecular profiling, including the analysis of epigenetic information. In combination with deep genome sequencing, the detection of SNVs can also be incorporated into current methods. SMD-seq offers a new direction for cancer research, with the integrated analysis of histology, transcriptome, and genome, it has the potential to enable a more comprehensive understanding of the tumorigenesis process and diagnosis.

## Materials and methods

### Pipeline of SMD-seq

#### 1. Preparation of tissue section

Tumor samples were collected from 4 patients who suffered various stages of OSCC at different ages and with different genders. All the biopsies were collected by protocols reviewed by and approved by the Ethics Committee of Peking University School and the Hospital of Stomatology (PKUSSIRB-201418116). Tumor samples diagnosed with OSCC were stored in DMEM supplemented with 10% (vol/vol) FBS at 4°C immediately after operation. Samples were stored in ice bucket and transported to lab within 30 min. The samples were then frozen in a cryostat (CM1950, Leica, Germany) at −20°C and were sectioned to 30 μm thin sections. The sections were placed onto polyethylene naphthalate (PEN) membrane slides (RNAase-Free, Leica, Germany). All sections were kept on ice before imaging.

#### 2. SRS imaging and laser microdissection in situ

Each slide was surveyed with SRS microscopy at Raman peaks 2,850 and 2,950 cm^−1^ immediately after sectioning. The 2 Raman peaks represent CH_2_ symmetric vibration and CH_3_ vibration, respectively. To obtain a fast switch between the 2 peaks, the wavelength of optical parametric oscillator (OPO) was set at 813 nm (corresponding approximately 2,901 cm^−1^) and switching to 809.8 nm and 816.4 nm by tuning the lyot filter in the cavity. Each image was scanned with a pixel dwelling time of 4 μs and a size of 640 × 640 pixels. To each image, a 2-frame Karlman filtering was applied. After above processing steps, the regions of cancerous cells were able to be identified by the experienced collaborating pathologist. A quick check at different focal planes was done before microdissection to make sure no big morphology change presenting within the 30 μm thickness. The post objective excitation power was 100 mW for pump and 40 mW for Stokes when imaging, and the pump (813 nm) power was increased to 180 to 190 mW to perform microdissection. To dissect an ROI, the laser repeatedly scanned 500 times along the border with a pixel dwelling time of 5 ms, hence effectively incised the PEN membrane from the glass slide, facilitating the cutting of ROIs. The dissected sample was manually transferred from slide to tube loaded with lysis buffer by RNase-free syringe needle. For each tissue section, we selected at least 1 dissected region of tumor and 2 dissected areas of similar size from normal tissues, one of them from the epithelium (the origin of this tumorigenesis) and the other from gland or muscle. To avoid the severe damage of RNA in the tissue sections, the whole procedure needs to be completed promptly.

#### 3. Transcriptome and genome sequencing

The dissected samples were put into lysis buffer [62] separately and immediately centrifuged at 13,000 rpm for 30 s. After lysis, each sample was equally divided into 2 aliquots for RNA-seq and genomic DNA sequencing, respectively. The protocol of RNA-seq was adapted from the pipeline of single-cell transcriptome analysis [62]. In brief, mRNA was reverse transcribed into first strand cDNA with polyT primer that has an anchor sequence. After other used primers were digested, polyA was added to the 3′ end of cDNA and second strand cDNA was formed and amplified with polyT primer with another anchor sequence by PCR. We employed degenerate oligonucleotide primed PCR (DOP-PCR) for amplifying the whole genome of each lysed tissue sample by the GenomePlex Single Cell Whole Genome Amplification Kit (WGA4-50RXN, Sigma-Aldrich, United States of America). For each sample, 50 ng of amplified genomic DNA and cDNA were used as the start amount of libraries preparation, separately. The pair-end sequencing libraries with approximately 300 bp insert size were constructed following the instructions of NEB Next Ultra DNA Library Prep Kit for Illumina (E7370, New England Biolabs, USA). Illumina HiSeq 2500 systems were used for sequencing.

### Stimulated Raman scattering (SRS) microscope

The home-built SRS system used a pump laser integrated OPO (picoEmerald, APE, Germany). It provided 2 spatially and temporally overlapped pulse trains, with the synchronized repetition rate of 80 MHz. One beam, fixed at 1,064 nm, was used as the Stokes light. The other beam, tunable from 780 to 990 nm, served as the pump light. The intensity of the Stokes beam was modulated at 20.2 MHz by a resonant electro-optical modulator (EOM). The overlapped lights were directed into an inverted multiphoton scanning microscope (FV1000, Olympus, Japan). The collinear laser beams were focused into the sample by a 20× objective (UPlanSAPO, NA 0.75, Olympus, Japan). Transmitted light was collected by a condenser (NA 0.9, Olympus, Japan). After filtering out the Stokes beam, the pump beam was directed onto a large area photo diode (FDS1010, Thorlabs, USA). The voltage from photo diode was sent into lock-in amplifier (HF2LI, Zurich Instruments, Switzerland) to extract the SRS signal. Image was reconstructed through software provided by manufacture (FV10ASW, Olympus, Japan).

### Image analysis

For 2-color SRS image, we applied a linear combination approach [63] on each field of view to convert the 2,850 and 2,950 cm^−1^ images into a reconstructed pseudo-color image (S1 Fig), in which we represented protein- and lipid-rich regions with blue and yellow, respectively. For texture analysis, all images were processed with the same procedure for HOG feature extraction. Dual color SRS images were resized to 80 × 80 pixels and subjected to Matlab (Mathwork, USA) built-in function “extractHOGFeature” to generate feature vectors. Hierarchical clustering was applied to extracted feature vector clustering in R, with built-in function “hclust” (“ward.D” method). Image correlation matrix was generated in R.

### Sequencing data analysis

Reproducibility of SMD-seq of these samples were validated by checking the Spearman correlation coefficients (r) of expressed genes (average r = 0.7) and reads count (average r = 0.7) between biological replicates of the same patients. Adaptor contamination and low-quality reads (phred quality <20) were discarded from the raw data. Only samples with coefficient of variation (CV) of reads count per 1 M bin <0.25 (genomic DNA) and gene number more than 6,000 (FPKM > 0.1, RNA) were kept for analysis. For RNA-seq data, TopHat (v2.0.10) were used for sequencing alignment. Reference genome assembly hg19 and gene annotation files were downloaded from UCSC Genome Browser. FPKM values used for analyses were generated by Cufflinks (v2.1.1), and Cuffdiff (v2.2.1) was used for gene expression levels comparison. Significantly different expressed genes between muscle, gland, epithelium, and cancer were selected under the criteria that *p* value < 0.05 and |log2 (fold change)| >1. Gene functional annotation was performed by The Database for Annotation, Visualization, and Integrated Discovery (DAVID) v6.717 [64]. The purity of tumor samples was estimated by ESTIMATE [65] with gene expression data. They were submitted to ESTIMATE for calculation of each score, then pooled and compared with the database calculated from TCGA HNSCC samples (*n* = 522) by the developer. Gene fusion analysis was carried out by FusionCatcher (v0.99.4a) [66] with 4 mapping tools (Bowtie, Bowtie2, BLAT, and STAR). Matched normal samples were used for each patient to exclude the fusion genes that are also found in normal samples. Under following situations, the fusion were discarded: (1) both fusion genes are mutual paralogs; (2) one or both of the fusion genes were pseudogene; (3) reported only by 1 mapping tool or reported by 2 mapping tools only once; (4) no known genes existed in between the fusion genes; and (5) the distance between both genes were less than 100 kbp. Under these criteria, 24 fusion genes were discovered with more than 10 paired reads spanning 2 different genes sequences. The circular diagram of fusion gene was generated by CIRCOS (v0.67–7) [67]. RNA-seq data were used for variant calling by GATK (v3.4–0) according to GATK Best Practices recommendations [68]. We performed duplicate removal, SplitNCigarReads, base quality score recalibration before SNP calling and filtered out SNPs by Fisher Strand values (FS > 30.0), Qual by Depth values (QD < 2.0), and sequencing depth passing the quality filter (DP < 10). Annotation of SNPs was performed by SnpEff (v4.0) [69]. Significantly mutated genes in HNSCC were inferred from COSMIC (Catalogue of Somatic Mutations in Cancer) and a comprehensive previous study [70]. Spearman correlation coefficient was computed between tissue samples by function “cor” in R. The unsupervised hierarchical clustering was performed by the function “pheatmap” of package “pheatmap” in R, and the method of measuring the distance in clustering columns was “manhattan”. The 3D PCA plot was generated by R package “scatterplot3d.”

The sequencing depth of DNA-seq was approximately 0.1×. Genomic DNA sequencing reads were mapped to reference genome by bowtie2 (v2.2.3) [71]. After duplication removal of mappable reads, the counts of aligned reads were calculated in each 1 M bin along the genome (Fig 4A and 4C–4E). For each bin, the read count of each tumor sample was normalized by sequencing depth and the median read count of all normal tissue samples, and the generated copy number went through segmentation by Circular Binary Segmentation [43] (the significance level was set as 0.05). The mean gene expression values of cancer samples within each segment were also calculated and normalized by mean expression values of normal samples that had corresponding qualified (CV < 0.25) gDNA reads (Fig 4C–4E). Function “pheatmap” in R was adopted for CNAs clustering (Fig 4B). GISTIC 2.030 was adopted to analyze the significantly reoccurring focal alterations for the gDNA segmented data.

## Supporting information

S1 Materials and methods Histologically-resolved multiomics enables precise molecular profiling of human intratumor heterogeneity. (DOCX)

S1 Fig Reconstruction process of dual-color SRS histology image. SRS, stimulated Raman scattering.
(TIF)

S2 Fig Dual-color SRS images of Warthin’s tumor and mucoepidermoid carcinoma. SRS, stimulated Raman scattering.
(TIF)

S3 Fig SRS images of epithelium and cancer for unsupervised hierarchical clustering. SRS, stimulated Raman scattering.
(TIF)

S4 Fig Correlation matrix of samples in 16 cancer samples and 16 epithelium samples. (TIF)

S5 Fig Images of cancer infiltrating muscle of P4. (TIF)

S6 Fig Localization of SRS subimage in corresponding HE-stained images. HE, hematoxylin–eosin; SRS, stimulated Raman scattering.
(TIF)

S7 Fig Characterization of microdissection of micro tissues. (TIF)

S8 Fig RNA preservation comparison between HE-stained and unstained sections. HE, hematoxylin–eosin.
(TIF)

S9 Fig Spearman correlation coefficients calculated between SMD-seq samples. SMD-seq, SRS microdissection and sequencing.
(TIF)

S10 Fig Unsupervised hierarchical clustering and gene annotation of enriched genes in different tissues. (TIF)

S11 Fig Gene expression levels of AKR1B10, FTH1, and FTL between cancer and epithelium samples from different patients. (TIF)

S12 Fig Immunofluorescence images to show the protein expression level of GSTP1. (TIF)

S13 Fig Keratin pearls in cancer nest of P3. (TIF)

S14 Fig Three-dimensional location of cancer nests of P4. (TIF)

S15 Fig Stromal content evaluation. (TIF)

S16 Fig The validation of gene fusion events by Sanger sequencing. (TIF)

S17 Fig Fusion events of intratumor ROIs. ROI, regions of interest.
(TIF)

S18 Fig Gene fusion events in sample P4S2E. (TIF)

S19 Fig Sequencing coverage across the whole genome. (TIF)

S20 Fig Genome-wide copy number distribution at different sampling rates. (TIF)

S21 Fig Comparison of unsupervised clustering of normalized reads count between Ginkgo and our methods. (TIF)

S22 Fig Copy number variation of autosomes from all cancer samples. (TIF)

S23 Fig Gene expression fold change of autosomes. (TIF)

S24 Fig Copy number variation and gene expression fold change of the same sectioned slice. (TIF)

S25 Fig Significant focal copy number alterations of all the cancer samples analyzed by GISTIC2.0. (TIF)

S26 Fig Significantly mutated genes in OSCC discovered by previous study and COSMIC. COSMIC, Catalogue of Somatic Mutations in Cancer; OSCC, oral squamous cell carcinoma.
(TIF)

S27 Fig Effect of image size and sample damage caused by laser. (TIF)

S1 Table Patients’ information and corresponding dissected tissues. (TIF)

S2 Table Summary of RNA-seq datasets. (TIF)

S3 Table Summary of genomic DNA sequencing datasets. (TIF)

S4 Table Overview of spatial transcriptomic methods. (TIF)

S1 Data Summary of genomic sequencing and RNA sequencing data analysis. (XLSX)

S2 Data Summary of numeric values presented in figures of main text and supporting information. (XLSX)

S3 Data Sanger sequencing results of fusion genes. (ZIP)

## Abbreviations:

CNA: copy number alteration
COSMIC: Catalogue of Somatic Mutations in Cancer
CV: coefficient of variation
DOP-PCR: degenerate oligonucleotide primed PCR
EMT: epithelial to mesenchymal transition
EOM: electro-optical modulator
HE: hematoxylin–eosin
HNSCC: head and neck squamous cell carcinoma
HOG: histogram of orientation gradient
LCM: laser capture microdissection
OPO: optical parametric oscillator
OSCC: oral squamous cell carcinoma
PCA: principal component analysis
PEN: polyethylene naphthalate
PLR: protein-to-lipid ratio
ROI: regions of interest
SMD-seq: SRS microdissection and sequencing
SRS: stimulated Raman scattering
TCGA: the Cancer Genome Atlas
