# Supplementary material for: Histologically resolved multiomics enables precise molecular profiling of human intratumor heterogeneity
Source: PLoS Biol. 2022 Jul 1;20(7):e3001699. doi: 10.1371/journal.pbio.3001699 (PMC9282480; doi:10.1371/journal.pbio.3001699)
Supplement: S1 Materials and methods — (DOCX) [file pbio.3001699.s001.docx]

***Supplementary Material***

Histologically-resolved multiomics enables precise molecular profiling of human intratumor heterogeneity

Tao Chen^1,2,†,[+]^, Chen Cao^1,†,[++]^, Jianyun Zhang^3,†^, Aaron Streets^1[+++}*^, Yanyi Huang^1,2,4,5,6,7^*, and Tiejun Li^3,^*

^1^ Biomedical Pioneering Innovation Center (BIOPIC), School of Life Sciences, Peking University, Beijing 100871, China.

^2^ College of Engineering, Peking University, Beijing 100871, China.

^3^ Department of Oral Pathology, Peking University School and Hospital of Stomatology, Beijing 100081, China.

^4^ Peking-Tsinghua Center for Life Sciences, Peking University, Beijing 100871, China.

^5^Beijing Advanced Innovation Center for Genomics (ICG), Peking University, Beijing 100871, China.

^6^ College of Chemistry and Molecular Engineering, Peking University, Beijing 100871, China.

^7^ Institute for Cell Analysis, Shenzhen Bay Laboratory, Guangdong 518132, China.

* To whom correspondence should be addressed: astreets@berkeley.edu (A.S.); yanyi@pku.edu.cn (Y.H.); litiejun22@vip.sina.com (T.L.)

† These authors contributed equally to this work.

[+] present address: Huffington Center on Aging, Baylor College of Medicine, Houston, TX 77030, USA

[++] present address: Lewis-Sigler Institute for Integrative Genomics, Princeton University, Princeton, NJ 08540, USA

[+++] present address: Department of Bioengineering, University of California, Berkeley, Berkeley, CA, 94720, USA

**Materials and methods**

*Large area imaging*

Image with large field of view was accomplished by multipoint time lapse function integrated in microscope software (FV10-ASW, Olympus, Japan). The outline determined by scanning and registering images along the boundary of ROI (region of interest). The software planned a scanning path according to the selected outline. Then, the stage automatically moved the sample to follow the planned path and acquired the image sequence with their spatial information. Each frame took ~21 s to obtain an image of 1024×1024 pixels. For the large field-of-view label-free histological image, two scanning sequences were required for lipid and protein channel.

*Immunofluorescence imaging*

Fresh tissues were embedded with OCT tissue-freezing medium, Frozen sections (5 μm thick) were blocked with a solution containing 2.5% bovine serum albumin for 30 minutes at 37 °C. Subsequently, sections were incubated with GSTP1 (3F2) Mouse mAB (1:200, Cell Signaling Technology) at 4 °C overnight. The secondary antibody, goat anti-mouse IgG-Cy3 (Invitrogen), was applied. Nuclei were stained with DAPI. Slides were washed on glycerol (1:1) for examination. Immunofluorescent signals were viewed using a confocal laser scanning fluorescent microscope (ZEISS LSM 5 EXCITER laser scanning microscope, Carl Zeiss MicroImaging, Oberkochen, Germany).

*PCR, cloning and transfection*

The primers used for amplifying fusion junction fragments from extracted cDNA were listed in Supplementary Table 2. PCR was performed with following cycles: 94°C 3 mins, 35 cycles of 94 °C 30 s, 60 °C 30 s, and 72 °C 30 s, followed by 72 °C 5 mins. The PCR products were run on 2% agarose gel and gel-purification with Zymoclean Gel DNA Recovery Kit (D4008, Zymo, USA). Recovered DNA were cloned into pMD18-T vector (D101A, Takara, Japan), and transfected to DH5α competent cells (CD201, Transgen, China). After incubation overnight in 37 °C, single clones were picked, and subjected to PCR (primers were listed in the following Table) verification and Sanger sequencing (Supplementary Figure 9).

Table. Primers used for amplifying the fusion sequence.

| Primer Name | Target Fusion Gene | Primer Sequences (5'-3') |
| --- | --- | --- |
| MK-F | MYH9-KRT14 | CTGCCTACCTGAAGCTGCG |
| MK-R |  | AGGACCTGCTCGTGGGTG |
| AL-F | AKT3-LRRC45 | AGCAGCAGCAGAGAATCCAA |
| AL-R |  | CTCTCCCTGTCCAGCAGC |
| RM-F | RAB3D-MTMR14 | GGCGCCTCTTTCTCAGGTCC |
| RM-R |  | ATCCCAGCAAAGATGGGGTGG |

*Comparison between H&E stained and unstained cryosections*.

Eight 30-μm-thick OSCC cryosections were prepared successively after snap-frozen (Fig S6). One of them was for H&E staining, and the other 7 were kept unstained. 12 ROIs of 3 tissue types (cancer, epithelium, and muscle) were micro-dissected from the H&E stained section and subjected to cDNA extraction, with 4 ROIs of each tissue type. For unstained sections, 4 ROIs of cancer area were micro-dissected from each section every 20 minutes (T0-T6), and followed by cDNA extraction. Fragments of 3 housekeeping genes (GAPDH, β-Actin and PPIA) were amplified for qPCR (Takara Bio SYBR Premix Ex Taq, Clonetech, Japan). The experiment was performed twice.

**Fig. S1** Pseudo color SRS histological image reconstruction process.

**Fig. S2** Dual-color SRS images of Watson tumor and mucoepidermoid carcinoma.

**Fig. S3** SRS images for unsupervised hierarchical clustering. 16 images of cancer samples (C#) and 16 images of epithelium samples (E#) are shown. Epithelium samples were visually inspected and manually adjusted with orientation.

**Fig. S4** Correlation matrix of samples in 16 cancer samples and 16 epithelium samples (Fig S3). Each dot represents the correlation coefficient of HOG features between two samples. Source data in Supporting information S2 Data Sheet 2.21.

**Fig. S5** The muscle near cancer nests of P4. a) typical SRS image of muscle tissue, presenting high protein content. b,c) two cancer nests of P4, presenting muscle, which has been infiltrated by cancer, as marked by red arrows.

**Fig. S6** Localization of SRS subimage in corresponding H&E stained image. The first row presented stitched SRS image and corresponding H&E, both in gray scale. Three areas were selected (white dashed boxes) for localization in H&E image. The second row showed the correlation map between HOG features of selected SRS subimage and H&E stitched image. The highlighted spot in correlation map is the localized reference point of the subimage. It is notable that the epithelia presented a line of high correlation, indicating much higher structure conservation of the normal tissue.

**Fig. S7** Characterization of micro-dissection of micro tissues. a) SRS image and H&E staining image after microdissection of the same epithelium microsample. The magnified micrograph showed the width of incision line, which is about 9 µm. b) the statistics of cell number in collected microtissues. c-e) SMD-seq micro-tissues overall sample purity, stromal and immune cell contamination compared with TCGA samples. Source data for panel b-e in Supporting information S2 Data Sheet 2.22-2.23.

**Fig. S8** RNA preservation comparison between H&E stained and unstained sections. a-b) qPCR Ct values of housekeeping genes, including beta-actin, GAPDH, and PPIA, in two batches of experiments. Each batch contained 7 different time points for unstained cancer sample. c) statistics showed the different between RNA preservation different before and after staining. Source data in Supporting information S2 Data Sheet 2.24.

**Fig. S9** Spearman correlation coefficients calculated between SMD-Seq samples. Each dot in (a-c) represents the FPKM value (FPKM > 0.01) of one gene by RNA-Seq. Reads mapped in each 1M bin were normalized against total sequencing depth in DNA-Seq as plotted in (d-f). Source data in Supporting information S2 Data Sheet 2.25-2.30.

**Fig. S10** Unsupervised hierarchical clustering and gene annotation of enriched genes in different tissues. Unsupervised hierarchical clustering of differently expressed genes in epithelium and cancer. Top 10 GO terms with P-value < 0.05 are shown. GO terms were ranked by logarithm P-value. Typical images, including SRS and H&E staining, of each tissue type were shown. Source data in Supporting information S2 Data Sheet 2.31.

**Fig. S11** Gene expression levels of AKR1B10, FTH1, FTL between cancer (C, orange) and epithelium samples (E, cyan) from different patients. Source data in Supporting information S2 Data Sheet 2.32.

**Fig. S12** Immunofluorescence images to show the protein expression level of GSTP1. Green channel is fluorophore linked to protein antibody, representing GSTP1 positive region. Blue channel is DAPI, representing the neucleic. Group1 and group2 were replicates. Scale bar is 100 μm.

**Fig. S13** Keratin pearls in cancer nests of P3, marked by red circles.

**Fig. S14** Three-dimensional locations of cancer nests of P4.

**Fig. S15** Stromal content evaluation. (a) UMAP distribution of ~6,000 malignant and non-malignant cells of HNSCC patients from Puram et al 2017. SMD-seq cancer samples were projected to this UMAP. (b) The percentage of most similar cells between SMD-Seq samples and single-cell HNSCC data in each cluster of (a) was calculated and shown in the heatmap. Color is coded by the percentage. Source data for panel a in Supporting information S2 Data Sheet 2.33.

**Fig. S16** The validation of gene fusion events by Sanger sequencing. (a) Fusion of MYH9 and KRT14. Black lines connect the fusion parts of two genes. (b) Fusion of AKT3 and LRRC45. Source data in Supporting information S3_Data.zip.

**Fig. S17** Fusion events of intra-tumor ROIs. Orange lines indicated fusion genes with at least 10 span pair reads, grey lines represented the other fusion genes. Source data in Supporting information S1_Data Sheet 1.4-1.7.

**Fig. S18** (a) Diagram of gene fusion events of P4S2E and its SRS image. The ribbons represent the fusion gene pairs. Fusion gene with more than 10 mapped span pair reads is shown in red, and oncogene involved fusion is colored in green. (b) Fusion of RAB3D and MTMR14. Source data in Supporting information S1_Data Sheet 1.8.

**Fig. S19** Sequencing coverage across the whole genome, numbers at the bottom represent the chromosome numbers.

**Fig. S20** Genome-wide copy number distribution at different sampling rates. a) Sequencing coverage across the whole genome, numbers at the bottom represent the chromosome numbers. Copy number per 1Mbp bin across the whole genome with different sampling rate of 0.001×, 0.005×, 0.01×, 0.05×, 0.09×, and 0.1× are plotted in the left diagram. Chromosome numbers were labeled at the bottom. Sampling rate of 1 equals the total number of DNA sequence that has been sequenced. The histogram at the right end of each scatter plot illustrates the distribution of copy number in autosomes and XY chromosomes. The data are fitted Gaussian distribution. b) Evaluation of the copy number in Fig. S20a. The bar plots with error bars (left Y axis) present mean and standard deviation of the copy number across whole genome at different sampling rate. The line plots (right Y axis) present the change of coefficient of variation as sampling rate increases. Source data in Supporting information S2 Data Sheet 2.34-2.35.

**Fig. S21** Comparison of unsupervised clustering of normalized reads count between Ginkgo and our methods. Ginkgo Both of them shows the similar clustering results in which samples from the same patient were clustered.

**Fig. S22** Copy number variation of autosomes from all cancer samples. Grey dots represent the normalized logarithm fold change, red and cyan lines demonstrate the segments along each chromosome calculated by CBS algorithm.

**Fig. S23** Gene expression fold change of autosomes. Magenta and cyan lines were mean gene expression value within each segment, which were calculated by CBS algorithm with normalized reads number per 1M bin.

**Fig. S24** Copy number variation and gene expression fold change of the same sectioned slice. (a)number variation and gene expression fold change of the same sections. (b)Normalized gene expression level of cancer samples. Mean gene expression levels were calculated in each 1M bin along the genome. The copy number of each bin and its corresponding gene expression fold change are plotted. Source data in Supporting information S2 Data Sheet 2.36-2.37.

**Fig. S25** Significant focal copy number alterations of all the cancer samples analyzed by GISTIC 2.0. Red and blue lines represented amplification and deletions peak regions, separately. Amplification or deletion regions with < 0.25 q value were annotated with possible driver genes which were also identified in RNA-Seq as differently expressed genes.

**Fig. S26** Significantly mutated genes in OSCC discovered by previous study and COSMIC. Red indicated the gene mutated in corresponding samples, blue demonstrated there was no SNP found.

**Fig. S27** Effect of image size and sample damage caused by laser. (a) The same field of view imaged with different image sizes of 512×512 and 1024×1024. Cropped region covering the area was shown to see the difference. The cellular profile (closed white dash line) was clearer in the 1024×1024 image crop. Scale bar is 200 μm. (b) laser induced sample damage. The same field of view was imaged twice. In the first image, a bright spot could be seen (white circle, pointed out by arrow). It was the primary damage position. In the second image, the primary spot turned into a spreading down damage zone (pointed by white arrow).

**Table S1** Patients information and corresponding dissected tissues. The first column shows 2-color SRS histological image, second column demonstrates the same tissue staining by H&E after laser dissection, and the last one represents a 5 μm thick H&E stained tissue which is next to the section for SRS imaging.

**Table S2** Summary of RNA-Seq datasets

**Table S3** Summary of genomic DNA sequencing datasets.

**Table S4** Overview of spatial transcriptomic methods

**S1 Data** Summary of genomic sequencing and RNA sequencing data analysis,

1.1 all genes, expression levels of all genes in all samples,

1.2 diff genes, expression levels of differentially expressed genes in all samples,

1.3 fusion genes summary, summary of gene fusion events in each patient,

1.4 P1 fusion genes, details of gene fusion events in patient 1,

1.5 P2 fusion genes, details of gene fusion events in patient 2,

1.6 P3 fusion genes, details of gene fusion events in patient 3,

1.7 P4 fusion genes, details of gene fusion events in patient 4,

1.8 P4S2E fusion genes, details of gene fusion events of one epithelial micro-tissue sample in patient 4,

1.9 GISTIC Gain, recurrent copy number gain among all cancerous micro-tissue samples,

1.10 GISTIC Loss, recurrent copy number loss among all cancerous micro-tissue samples.

1.11 Fusion_CNV regions, summary of gene fusion events and copy number gain/loss.

**S2 Data** Summary of numeric values presented in figures of main text and supporting information. The data presented in each sheet are shown as,

2.1 Fig. 2c, number of detected genes in each micro-tissue sample,

2.2 Fig. 2d, coordinates of first three principal components of each micro-tissue sample after PCA analysis,

2.3 Fig. 2e, z-scores of differentially expressed genes of each micro-tissue sample

2.4 Fig. 3a, gene expression level (FPKM) of genes GSTP1, KRT13, KRTDAP, and KLK8 in each micro-tissue sample,

2.5 Fig. 4b, normalized reads count across the chromosomes of each cancerous micro-tissue,

2.6 Fig. 4c, normalized reads count and mean expression values of chromosome 2 in micro-tissue sample P1S3C,

2.7 Fig. 4d, normalized reads count and mean expression values of chromosome 11 in micro-tissue sample P1S1C,

2.8 Fig. 4e, normalized reads count and mean expression values of chromosome 18 in all cancerous micro-tissue,

2.9 Fig. 4f, gene expression fold changes at different copy numbers across the genome of all cancerous micro-tissue,

2.10 Fig. 4g-config, software settings used to generate Fig. 4g,

2.11 Fig. 4g-fusion1M, gene fusion events across the transcriptome of all cancerous micro-tissue samples,

2.12 Fig. 4g-gainloss, copy number loss and gain across genome of all cancerous micro-tissue samples,

2.13 Fig. 4g-histP1S1C, histogram of copy number loss and gain in micro-tissue sample P1S1C,

2.14 Fig. 4g-histP1S3C, histogram of copy number loss and gain in micro-tissue sample P1S3C,

2.15 Fig. 4g-histP1S4C, histogram of copy number loss and gain in micro-tissue sample P1S4C,

2.16 Fig. 4g-histP2S2C, histogram of copy number loss and gain in micro-tissue sample P2S2C,

2.17 Fig. 4g-histP3S4C, histogram of copy number loss and gain in micro-tissue sample P3S4C,

2.18 Fig. 4g- histP3S5C, histogram of copy number loss and gain in micro-tissue sample P3S5C,

2.19 Fig. 4g-histP4S1C, histogram of copy number loss and gain in micro-tissue sample P4S1C,

2.20 Fig. 4g-histP4S2C, histogram of copy number loss and gain in micro-tissue sample P4S2C,

2.21 Fig. S4, spearman correlation matrix between HOG features of 16 cancer samples and 16 normal samples SRS images,

2.22 Fig. S7b, cell numbers of each different micro-tissue types,

2.23 Fig. S7c-e, the StromalScore, ImmuneScore and ESTIMATEScore of all micro-tissue samples and TCGA OSCC samples,

2.24 Fig. S8, Ct values of three house-keeping genes, GAPDH, ACTB, and PPIA in unstained and stained samples,

2.25 Fig. S9a, gene expression levels of cancerous micro-tissue samples of patient1,

2.26 Fig. S9b, gene expression levels of cancerous micro-tissue samples of patient2,

2.27 Fig. S9c, gene expression levels of cancerous micro-tissue samples of patient3,

2.28 Fig. S9d, normalized reads per 1M bin of cancerous micro-tissue samples of patient1,

2.29 Fig. S9e, normalized reads per 1M bin of cancerous micro-tissue samples of patient2,

2.30 Fig. S9f, normalized reads per 1M bin of cancerous micro-tissue samples of patient3,

2.31 Fig. S10, GO terms and p-value of different tissues,

2.32 Fig. S11, expression levels of genes AKR1B10, FTH1, and FTL in all cancerous and epithelial micro-tissue samples,

2.33 Fig. S15a, UMAP coordinates of each micro-tissue samples and HNSCC single cells (Puram et al 2017),

2.34 Fig. S20a, normalized reads count of P4S1E at different sampling rate,

2.35 Fig. S20b, statistics of normalized reads count of P4S1E at different sampling rate,

2.36 Fig. S24a, copy numbers and gene expression fold changes of each cancerous micro-tissue sample,

2.37 Fig. S24b, gene expression fold changes at different copy number in each cancerous sample.

**S3 Data** Sanger sequence of fused genes, AKT3 and LRRC45, MYH9 and KRT 14, RAB3D and MTMR14.
