# Supplementary figures and images for: Histologically resolved multiomics enables precise molecular profiling of human intratumor heterogeneity
Source: PLoS Biol. 2022 Jul 1;20(7):e3001699. doi: 10.1371/journal.pbio.3001699 (PMC9282480; doi:10.1371/journal.pbio.3001699)

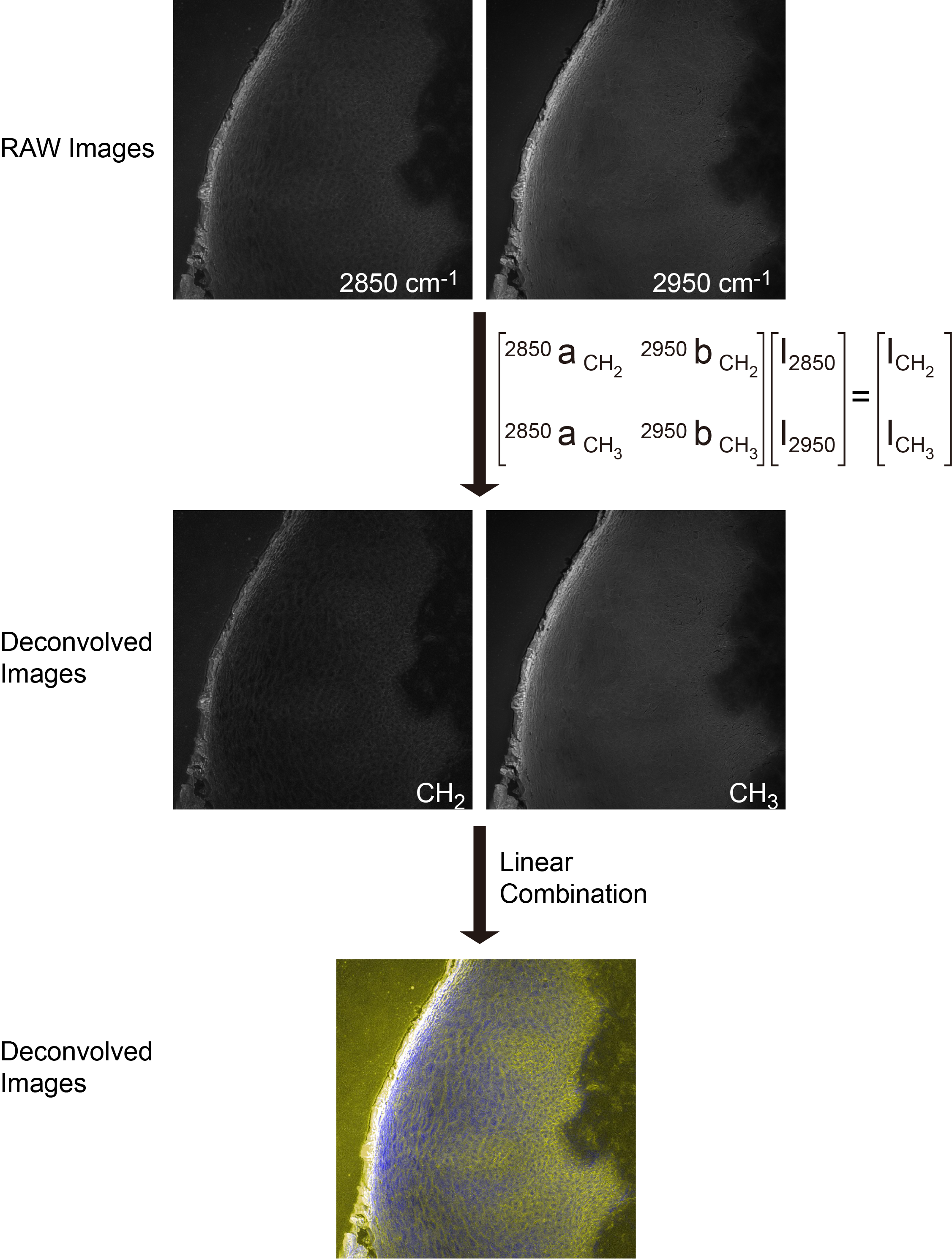

Supplement: S1 Fig — SRS, stimulated Raman scattering. (TIF) [file pbio.3001699.s002.tif]

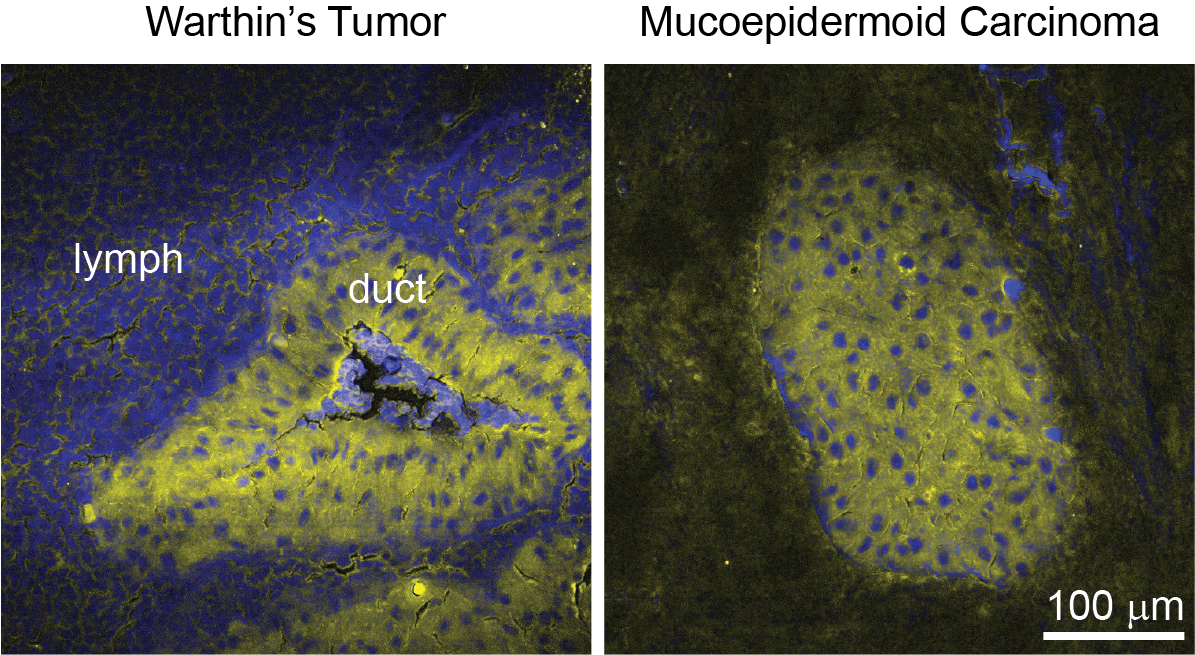

Supplement: S2 Fig — SRS, stimulated Raman scattering. (TIF) [file pbio.3001699.s003.tif]

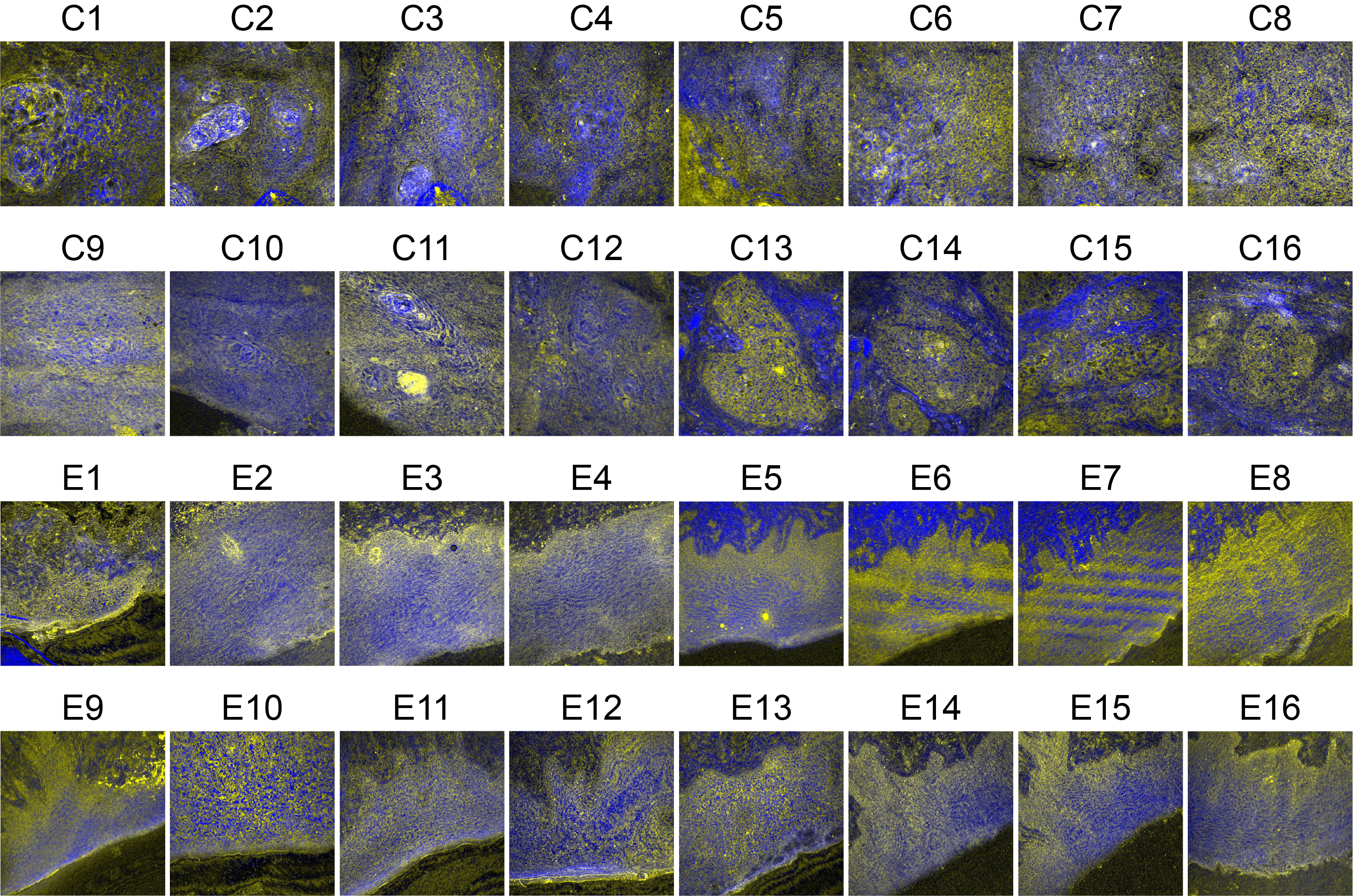

Supplement: S3 Fig — SRS, stimulated Raman scattering. (TIF) [file pbio.3001699.s004.tif]

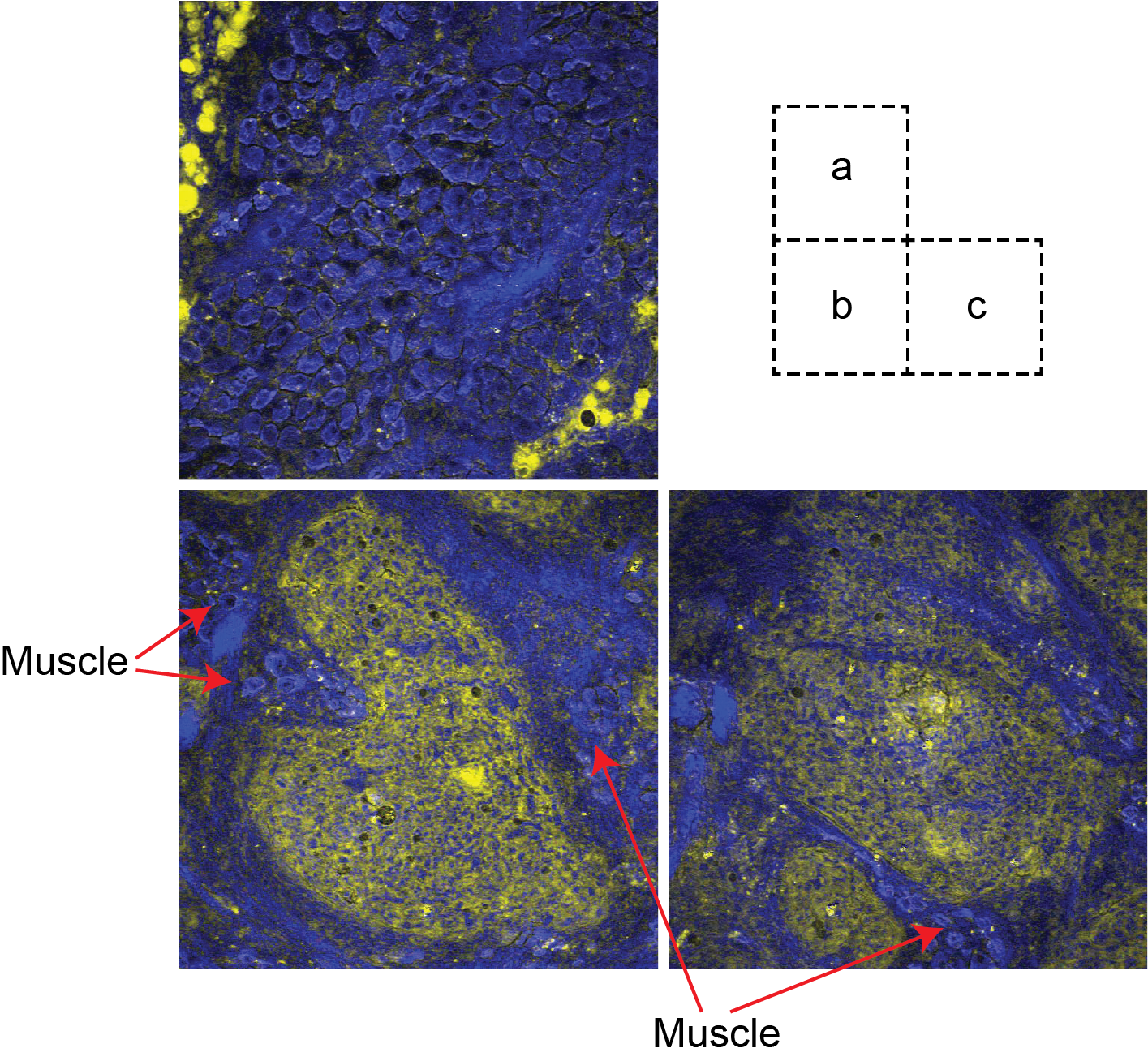

Supplement: S5 Fig — (TIF) [file pbio.3001699.s006.tif]

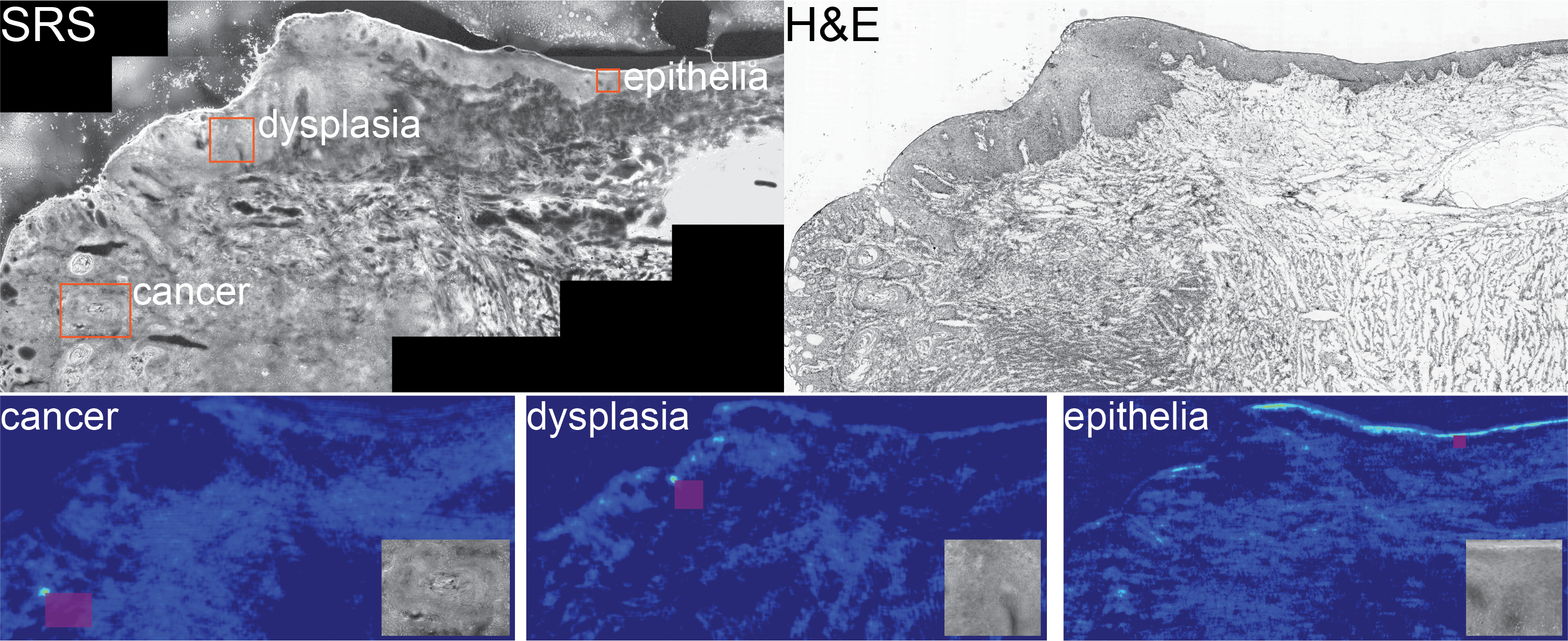

Supplement: S6 Fig — HE, hematoxylin–eosin; SRS, stimulated Raman scattering. (TIF) [file pbio.3001699.s007.tif]

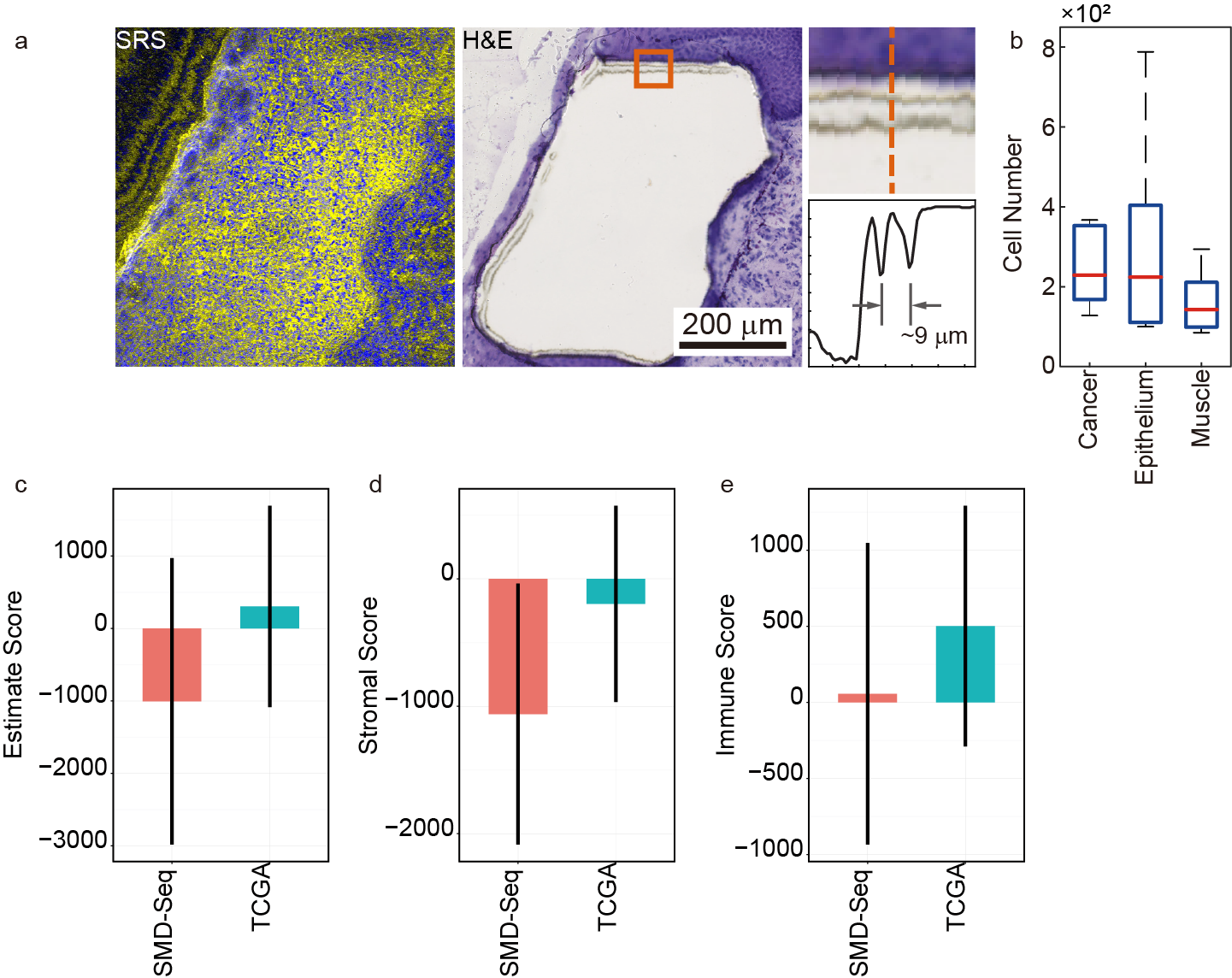

Supplement: S7 Fig — (TIF) [file pbio.3001699.s008.tif]

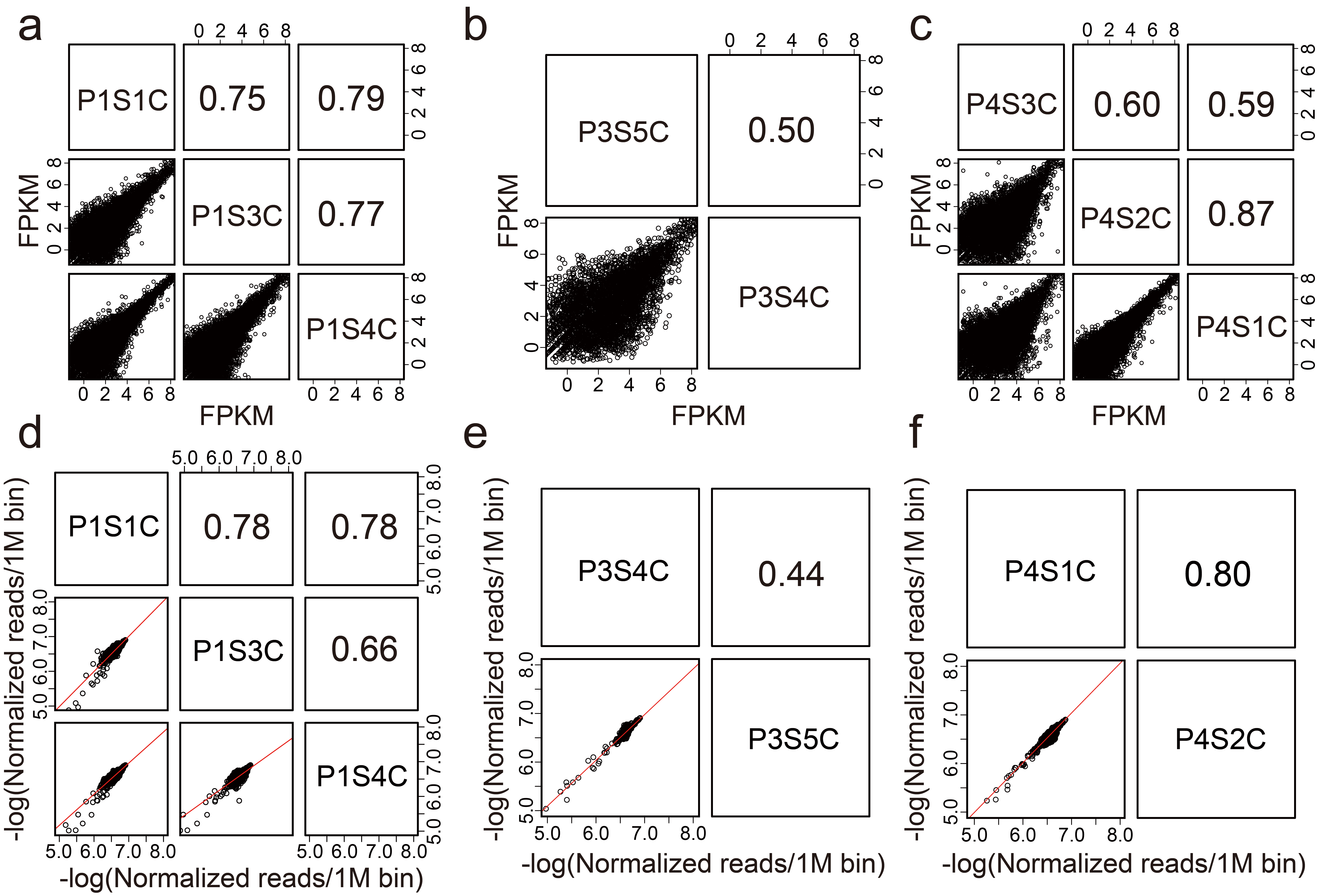

Supplement: S9 Fig — SMD-seq, SRS microdissection and sequencing. (TIF) [file pbio.3001699.s010.tif]

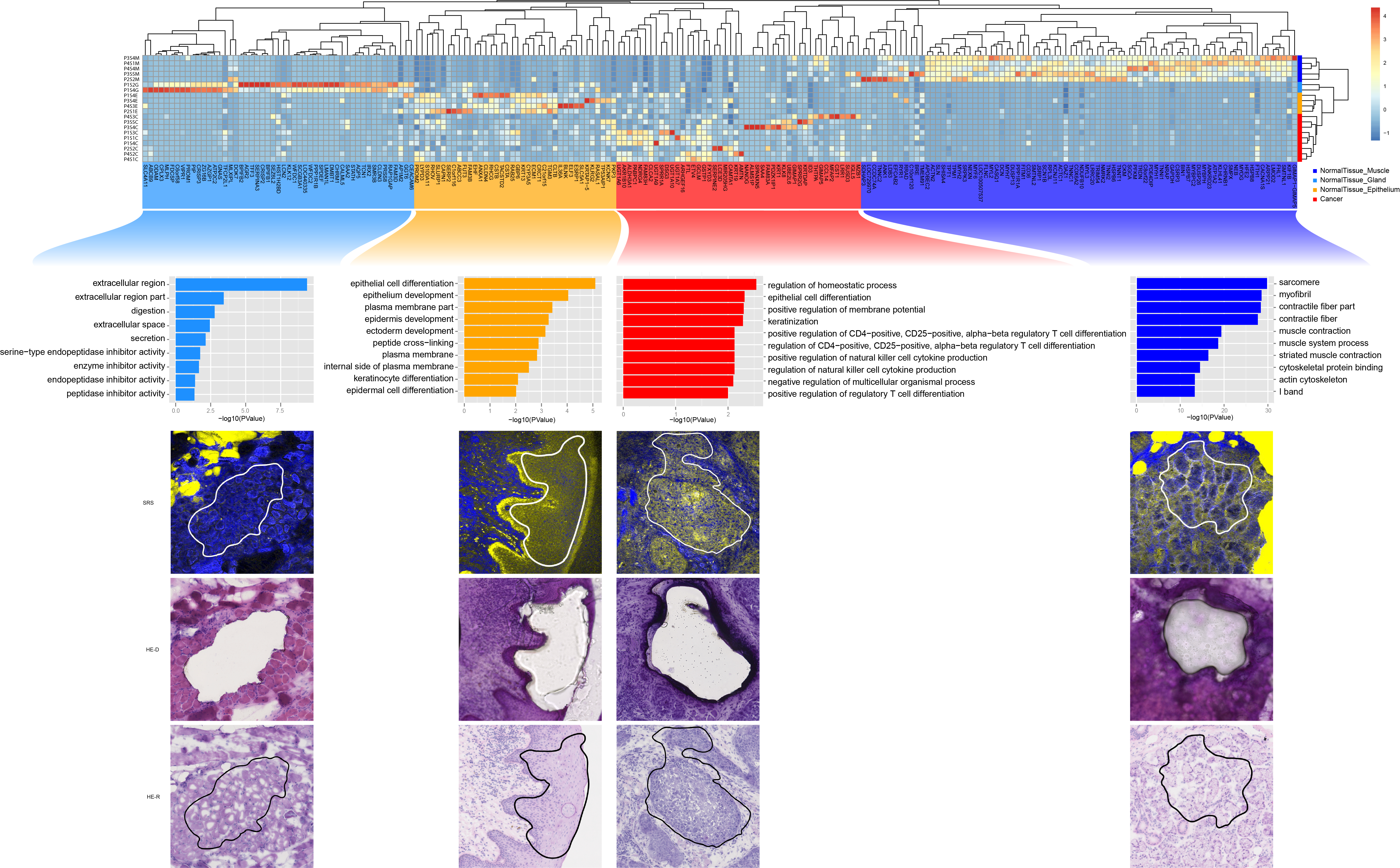

Supplement: S10 Fig — (TIF) [file pbio.3001699.s011.tif]

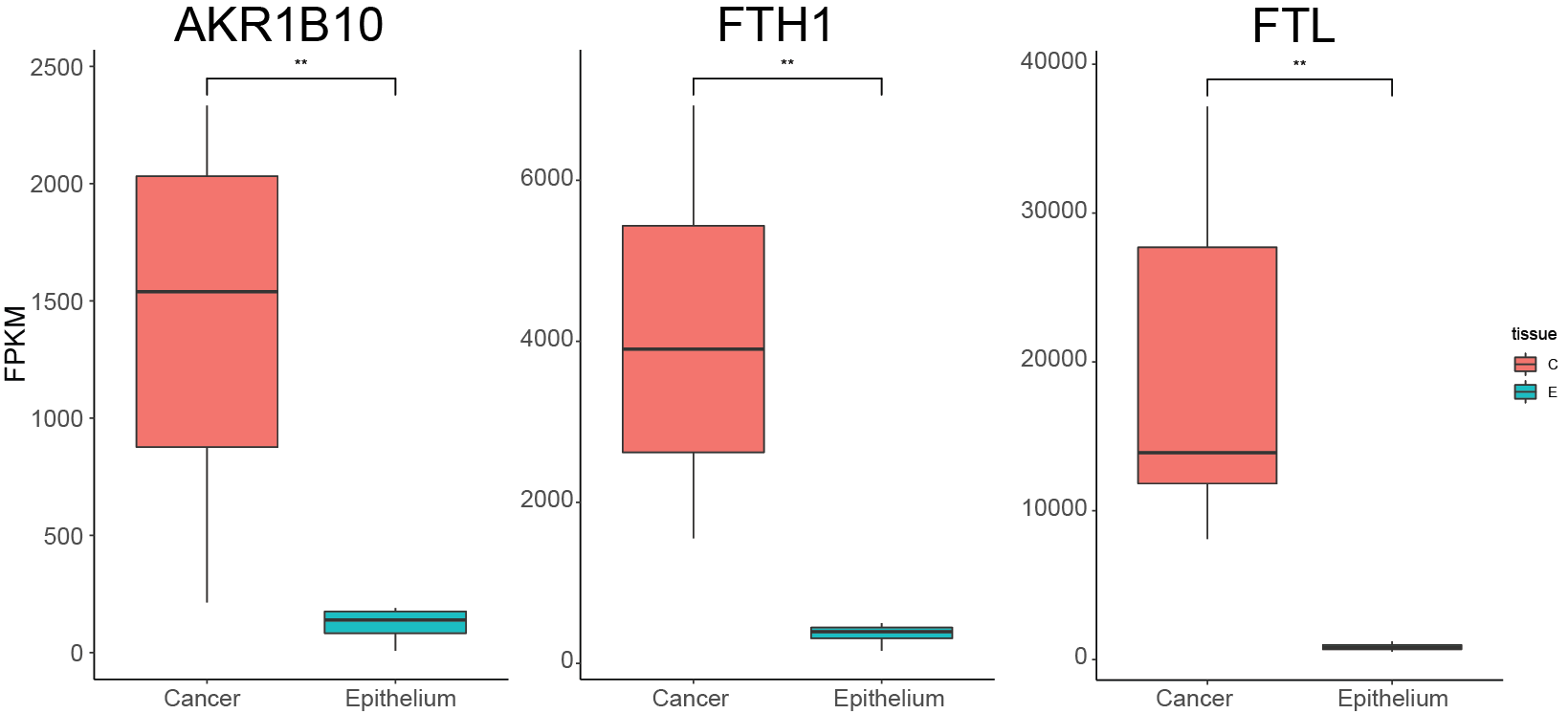

Supplement: S11 Fig — (TIF) [file pbio.3001699.s012.tif]

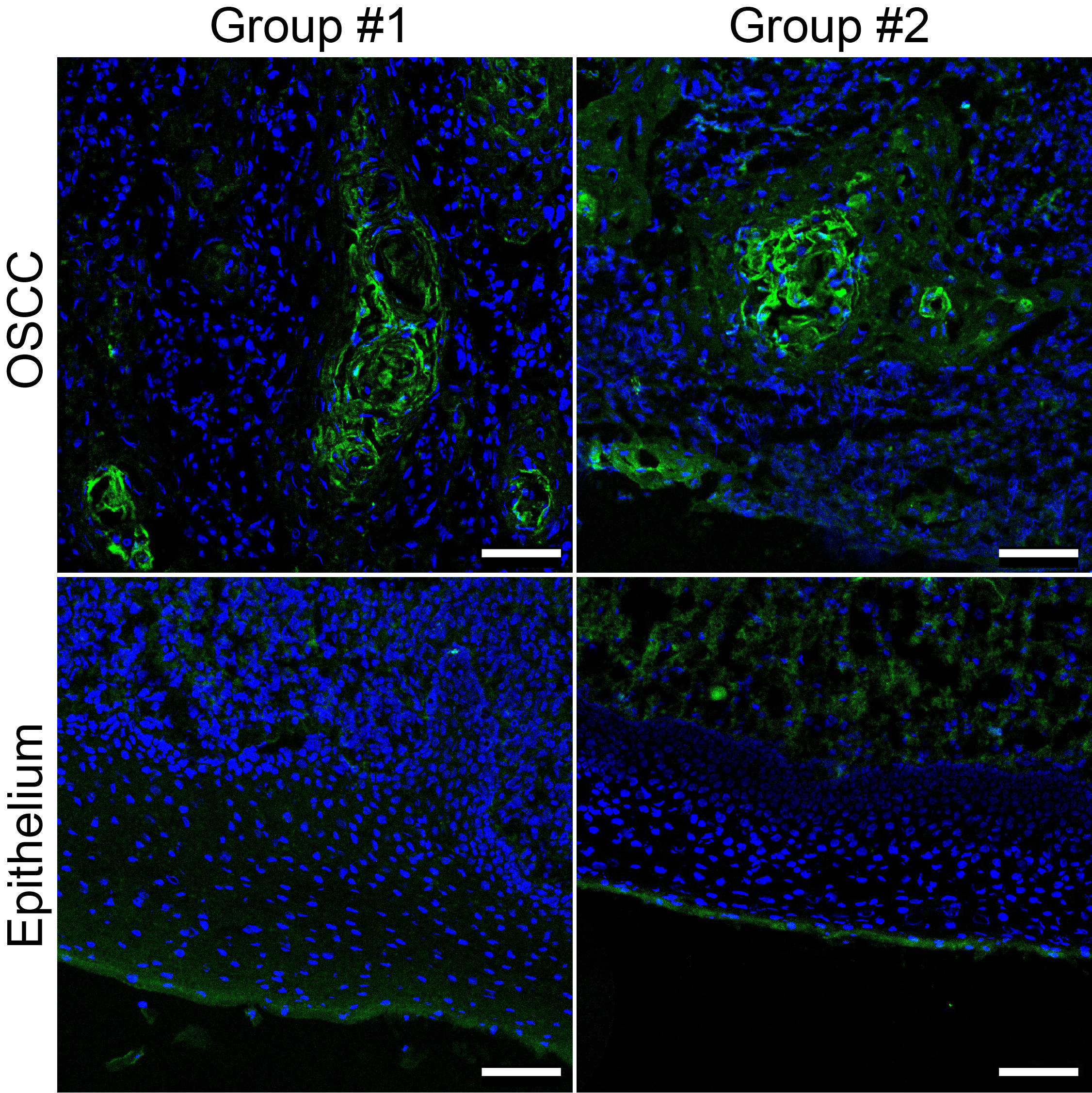

Supplement: S12 Fig — (TIF) [file pbio.3001699.s013.tif]

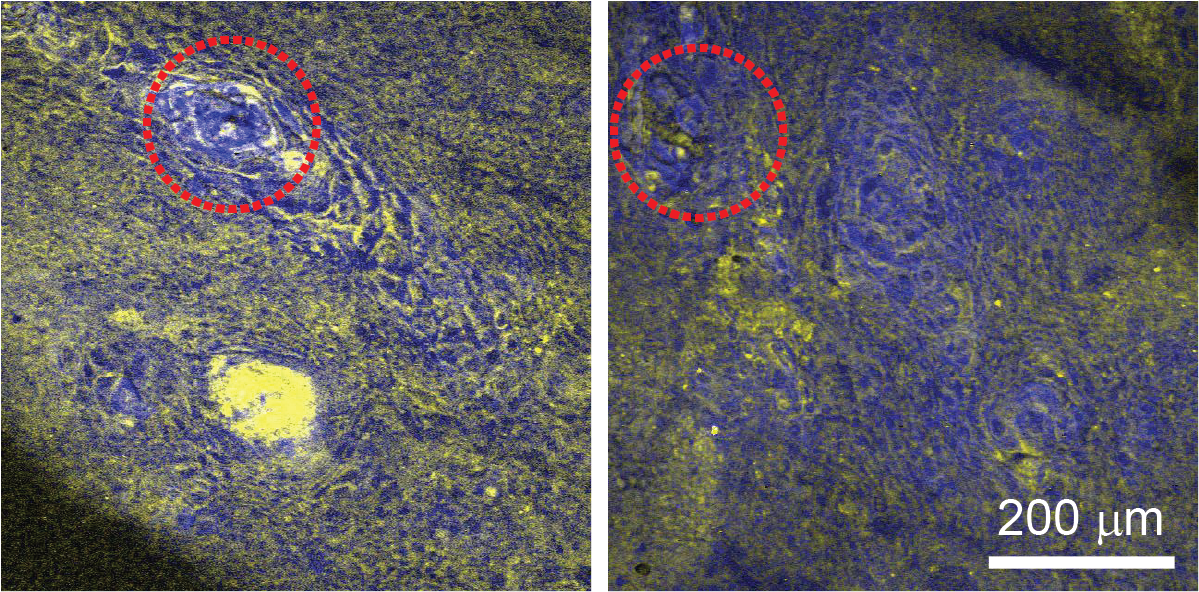

Supplement: S13 Fig — (TIF) [file pbio.3001699.s014.tif]

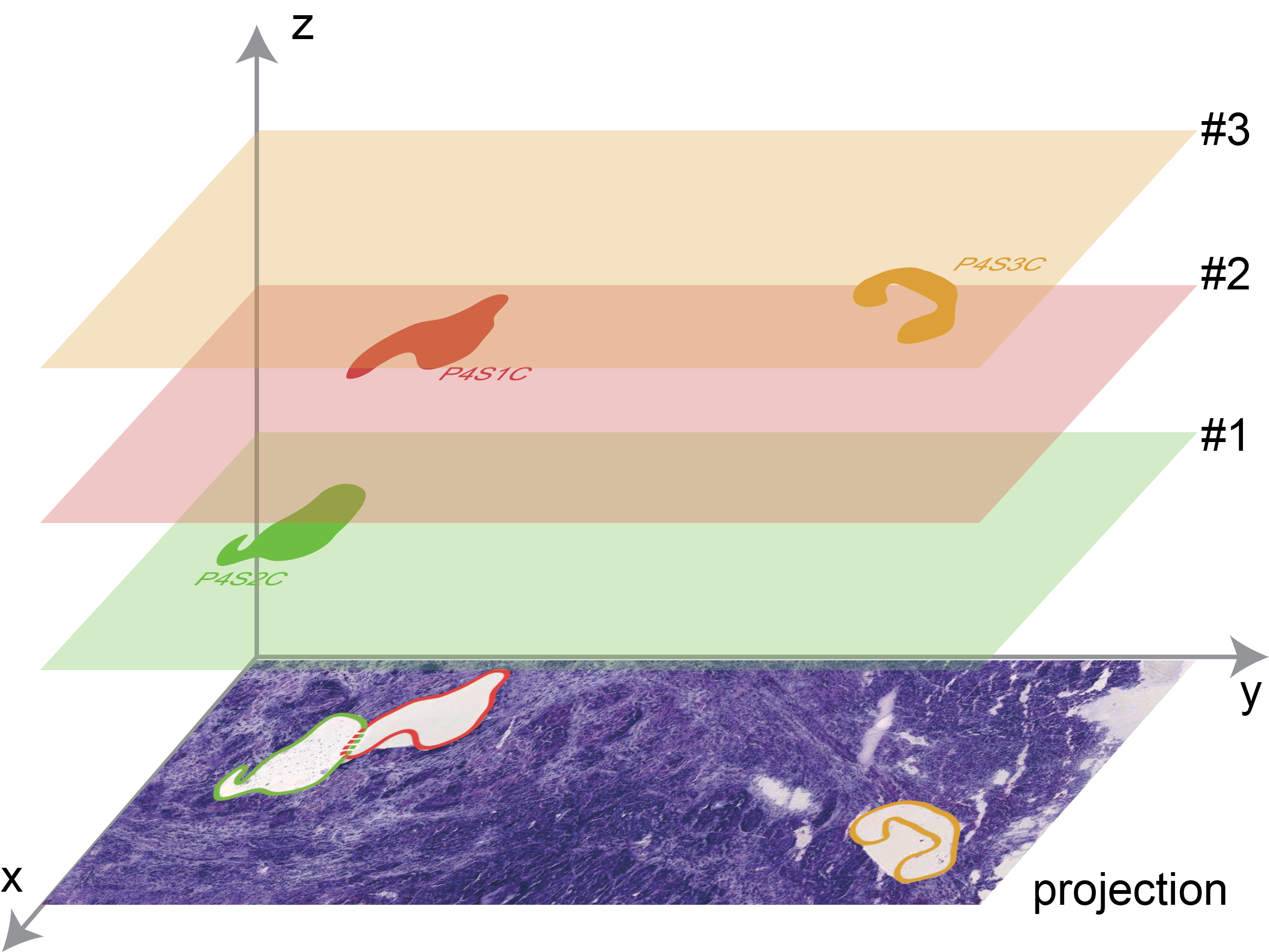

Supplement: S14 Fig — (TIF) [file pbio.3001699.s015.tif]

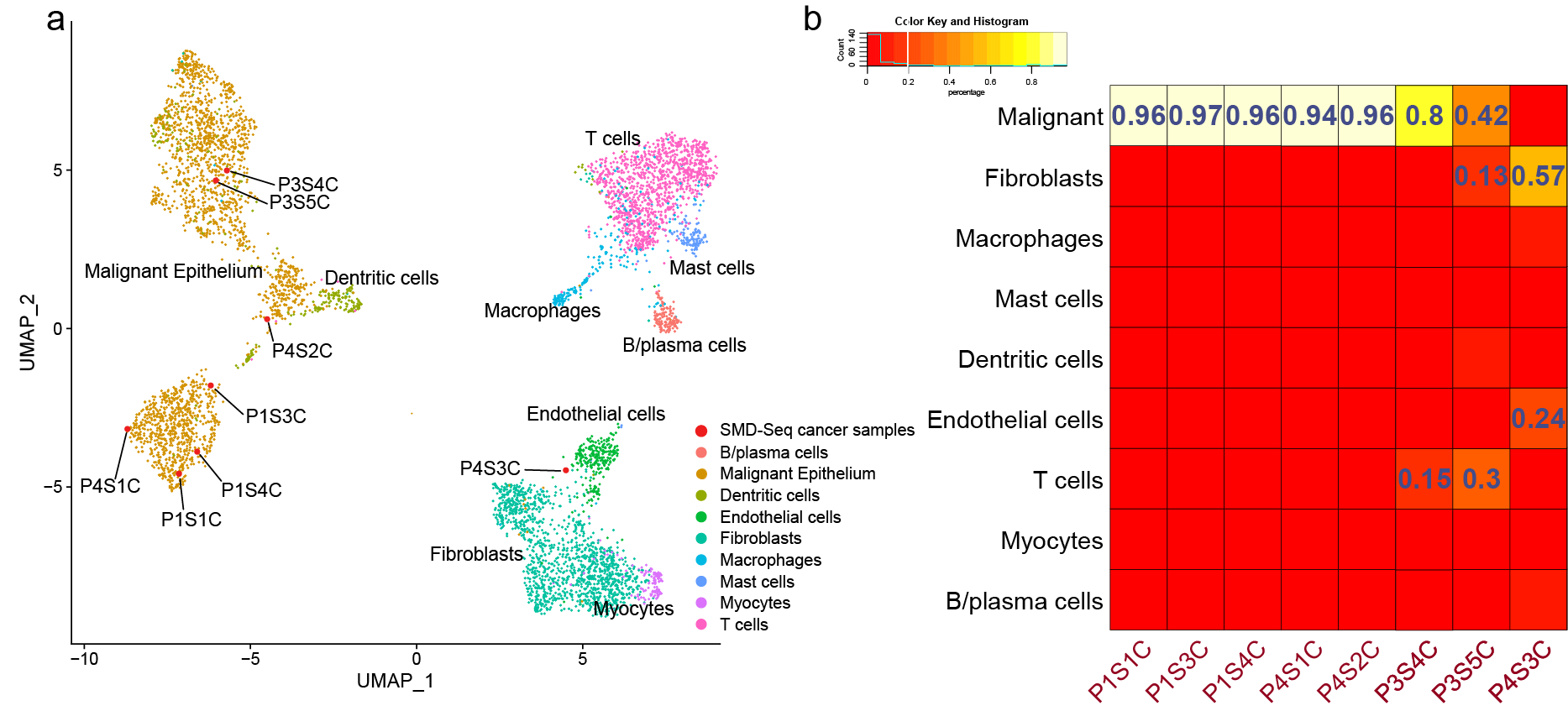

Supplement: S15 Fig — (TIF) [file pbio.3001699.s016.tif]

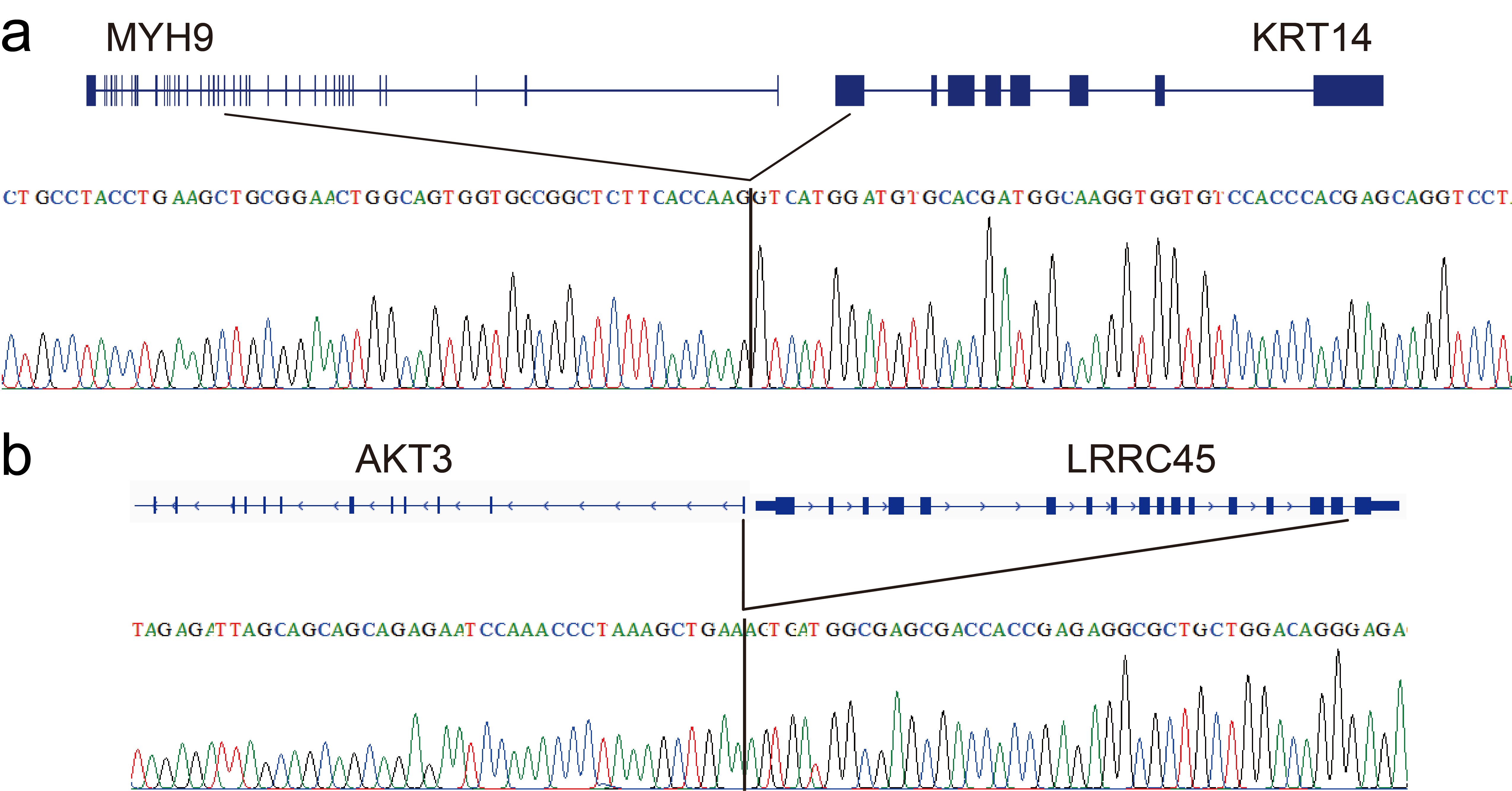

Supplement: S16 Fig — (TIF) [file pbio.3001699.s017.tif]

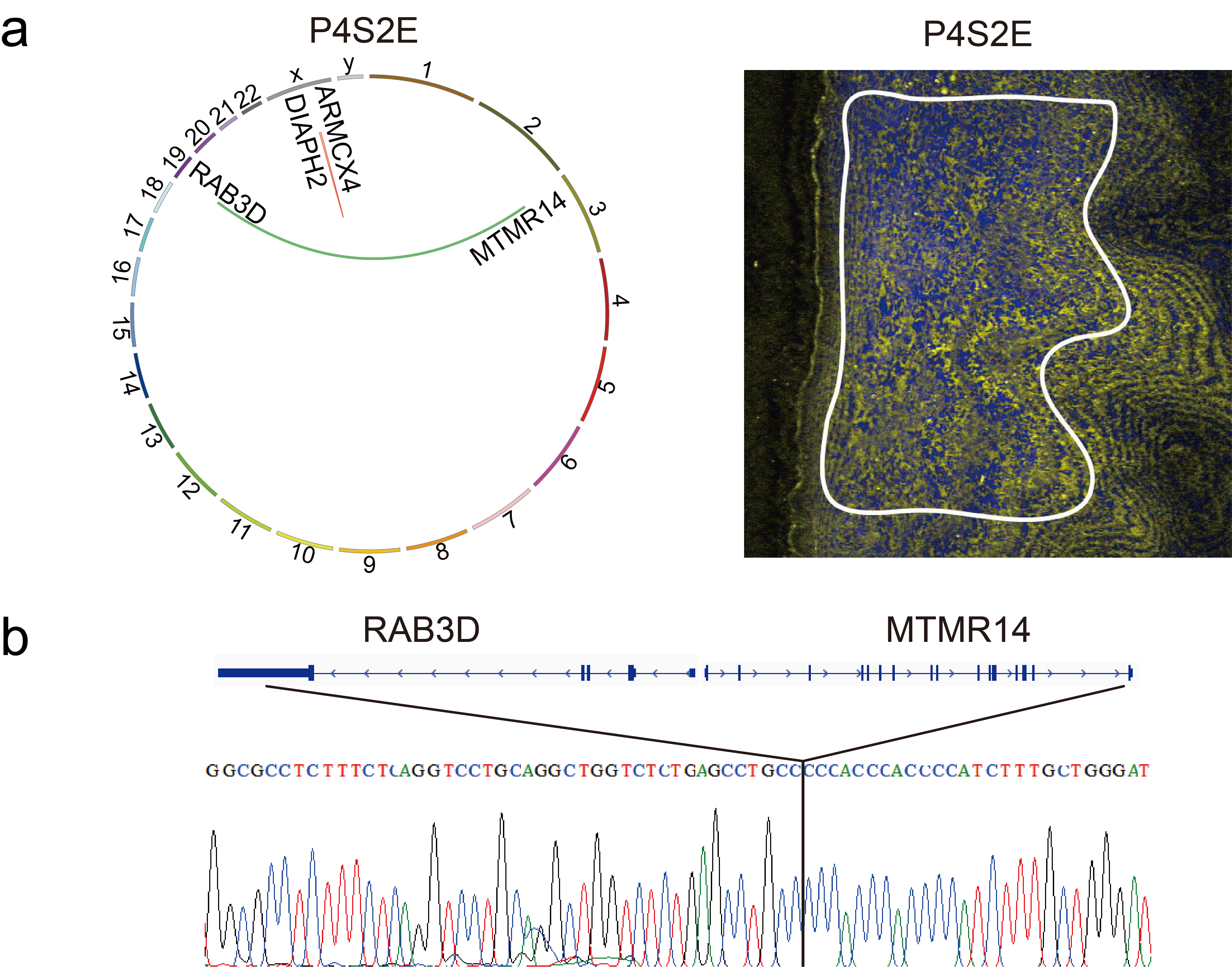

Supplement: S18 Fig — (TIF) [file pbio.3001699.s019.tif]

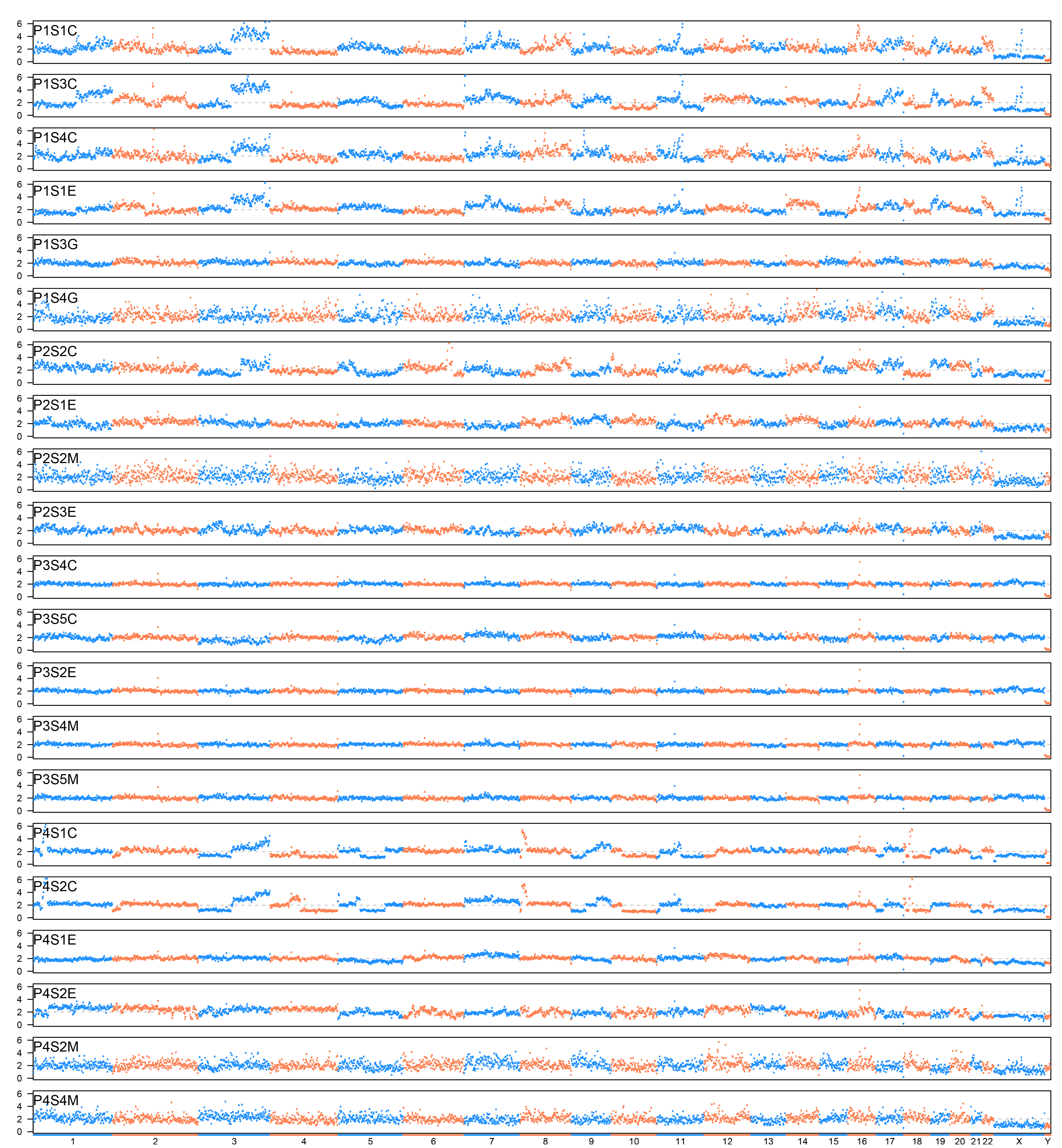

Supplement: S19 Fig — (TIF) [file pbio.3001699.s020.tif]

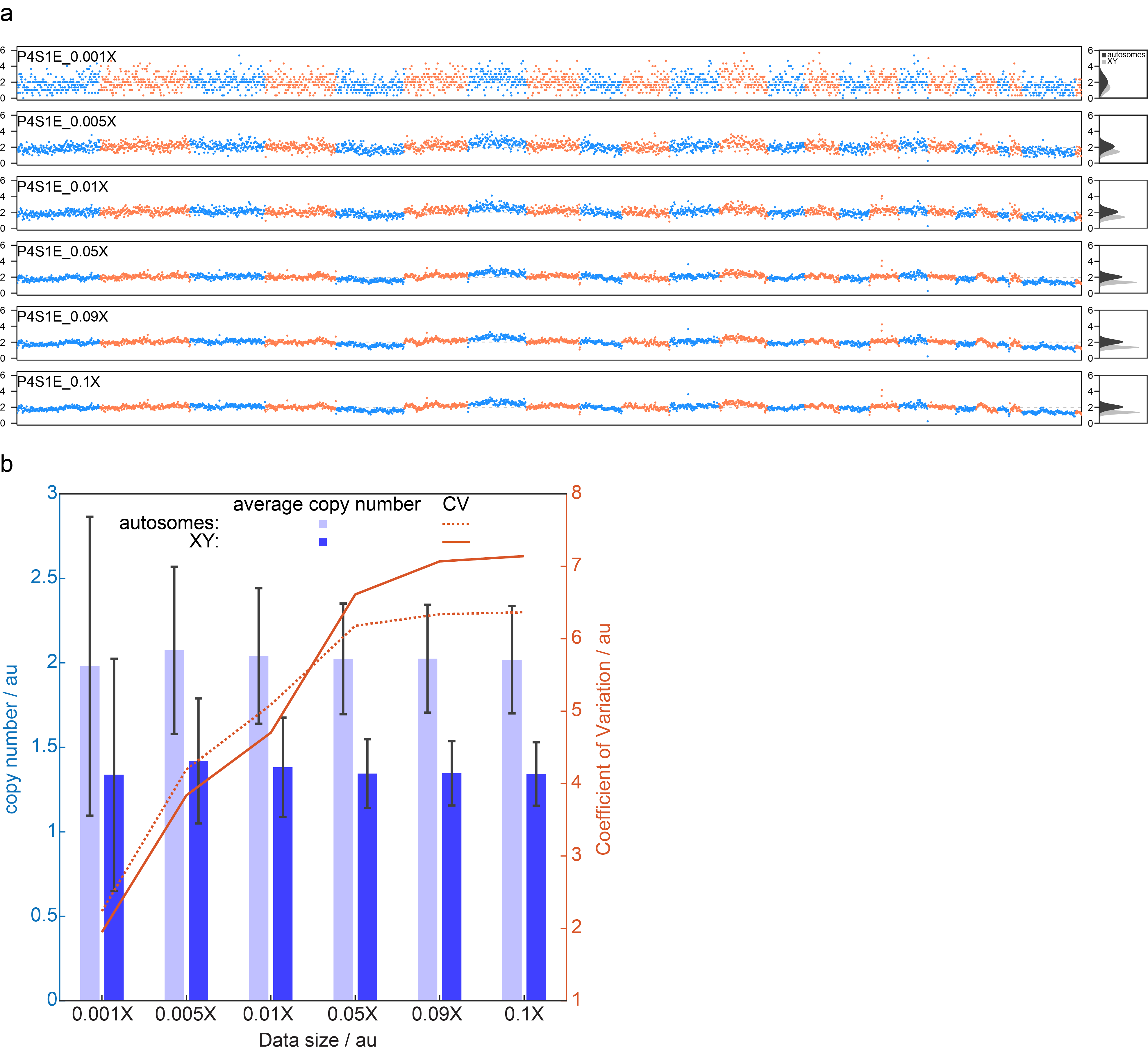

Supplement: S20 Fig — (TIF) [file pbio.3001699.s021.tif]

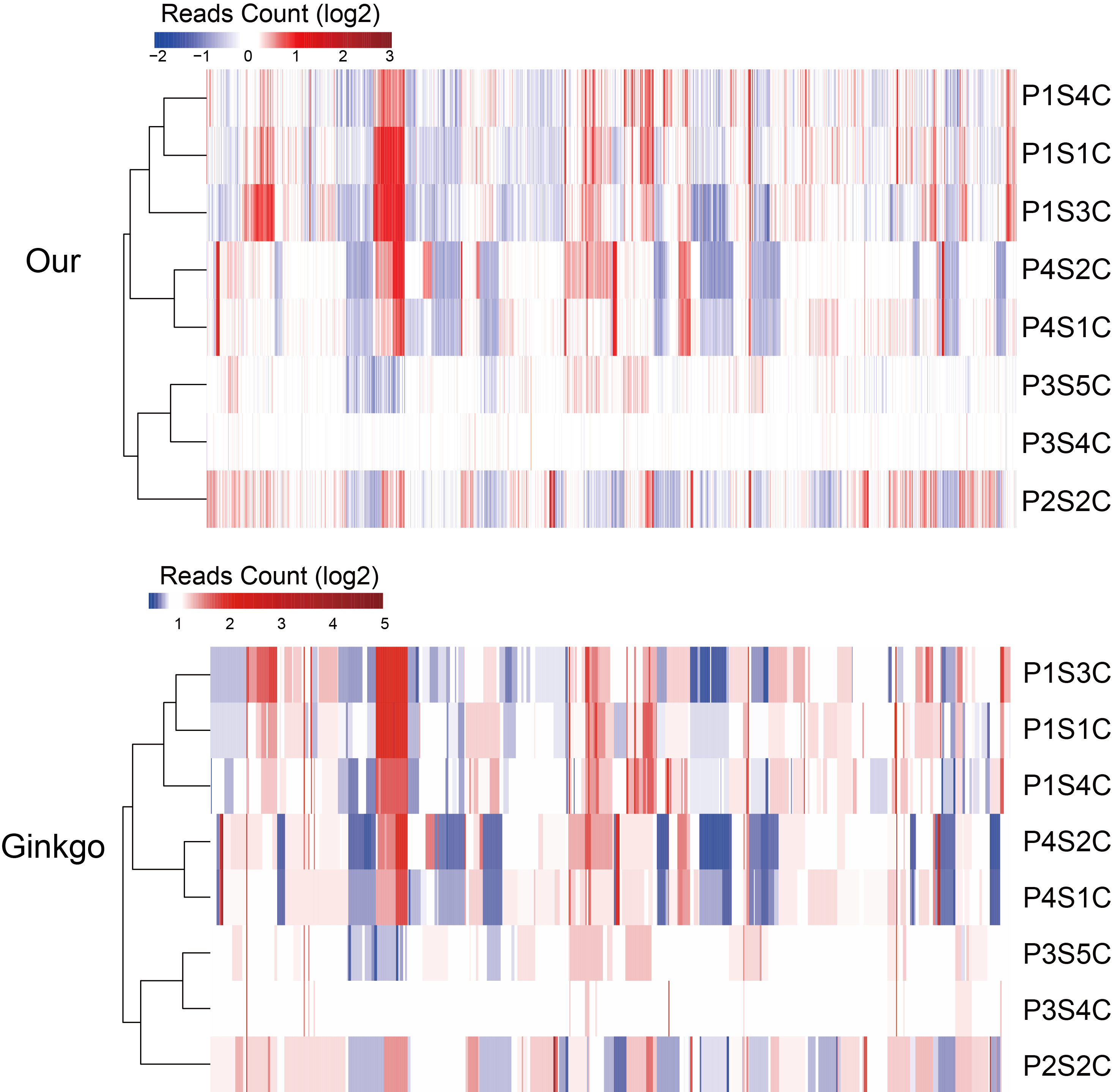

Supplement: S21 Fig — (TIF) [file pbio.3001699.s022.tif]

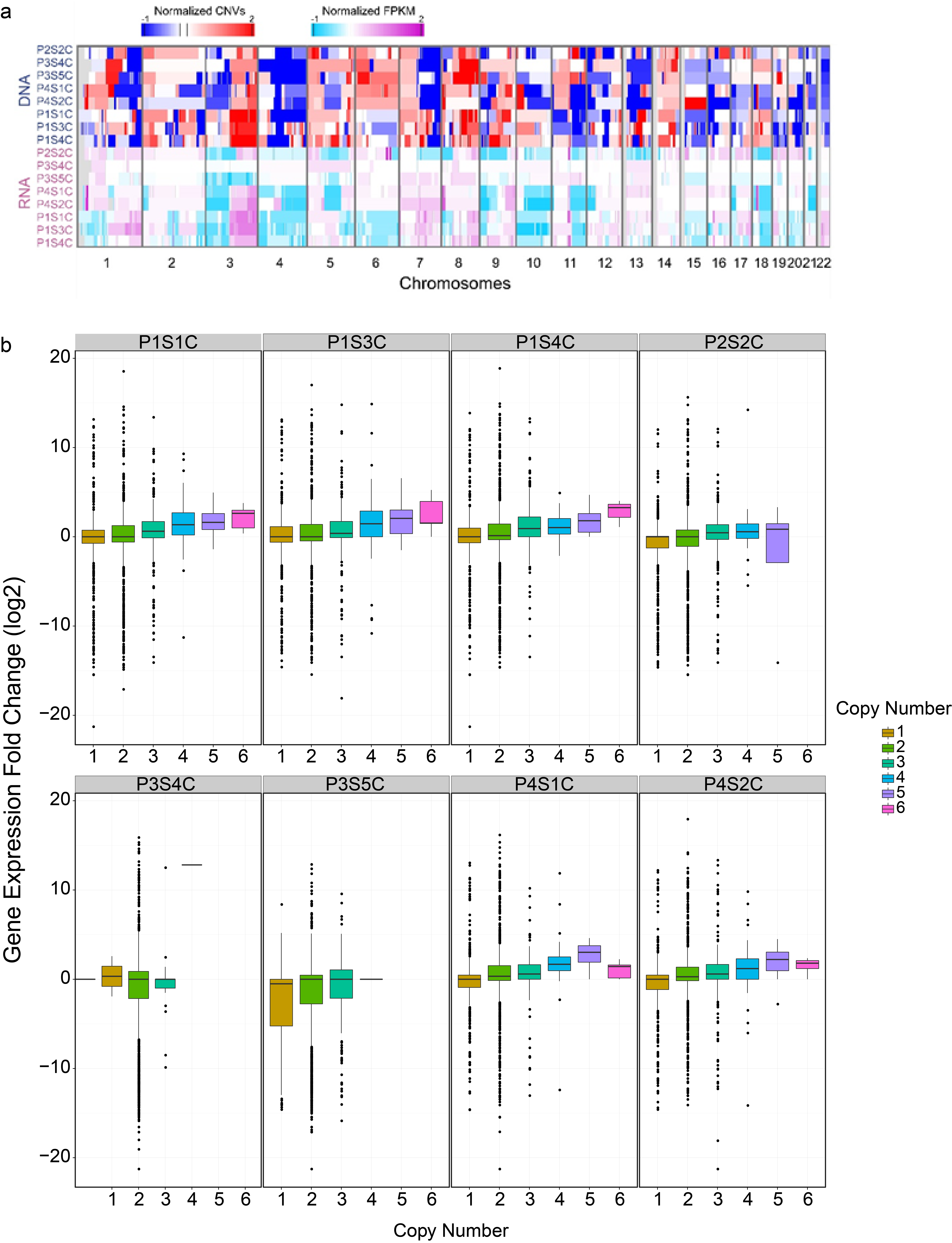

Supplement: S24 Fig — (TIF) [file pbio.3001699.s025.tif]

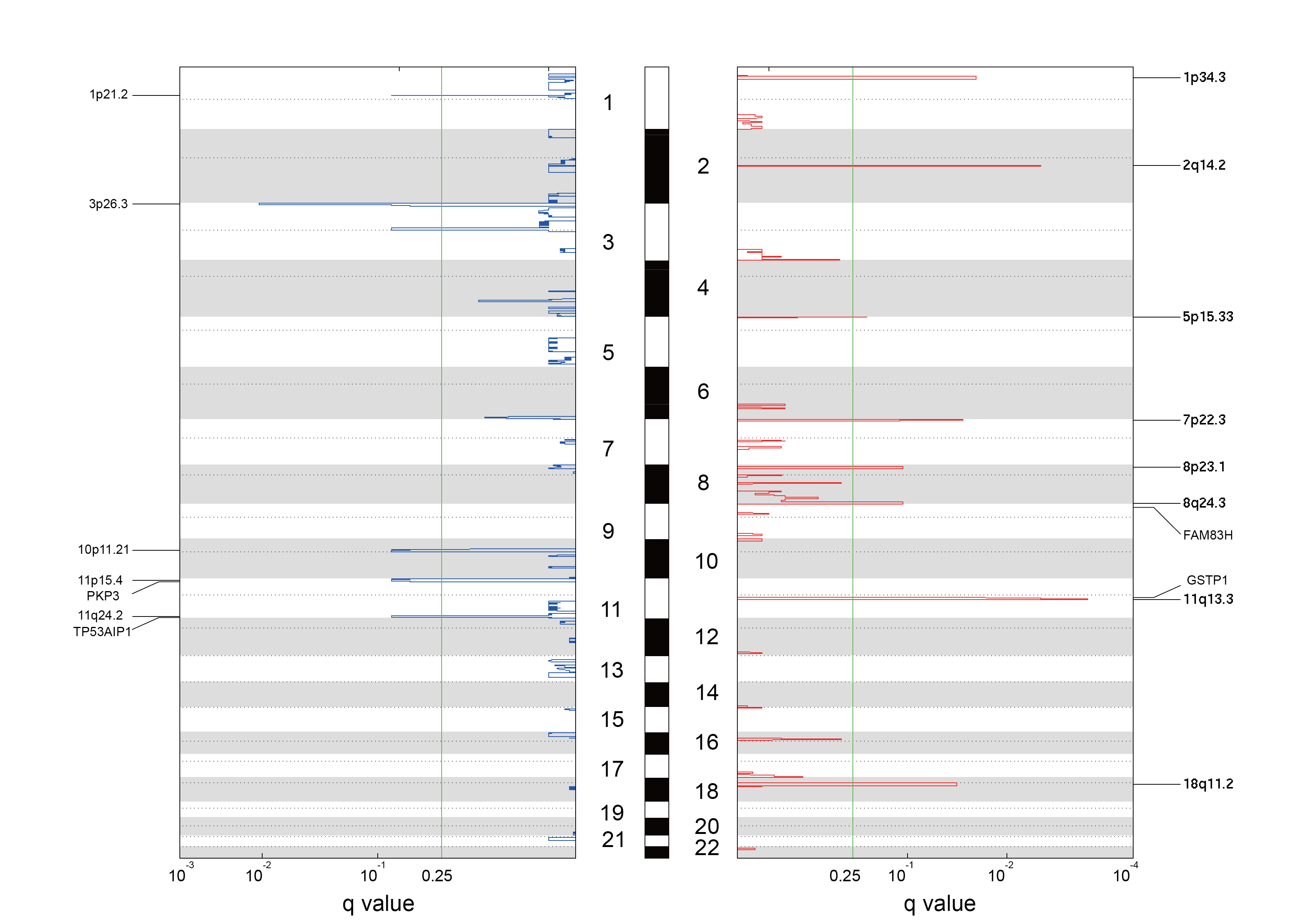

Supplement: S25 Fig — (TIF) [file pbio.3001699.s026.tif]

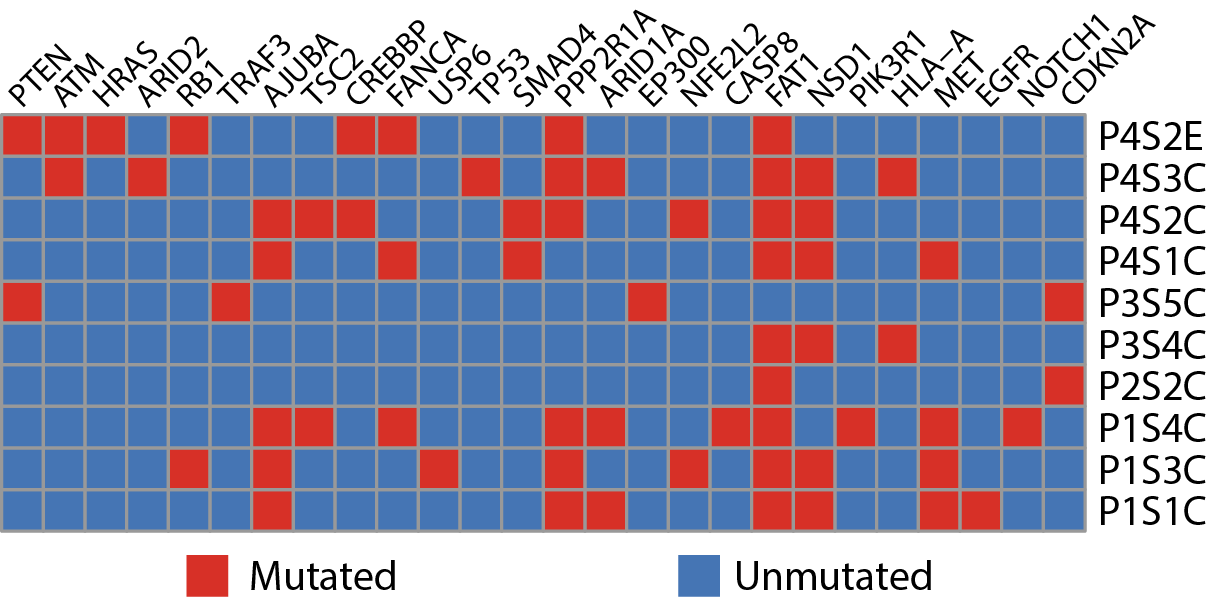

Supplement: S26 Fig — COSMIC, Catalogue of Somatic Mutations in Cancer; OSCC, oral squamous cell carcinoma. (TIF) [file pbio.3001699.s027.tif]

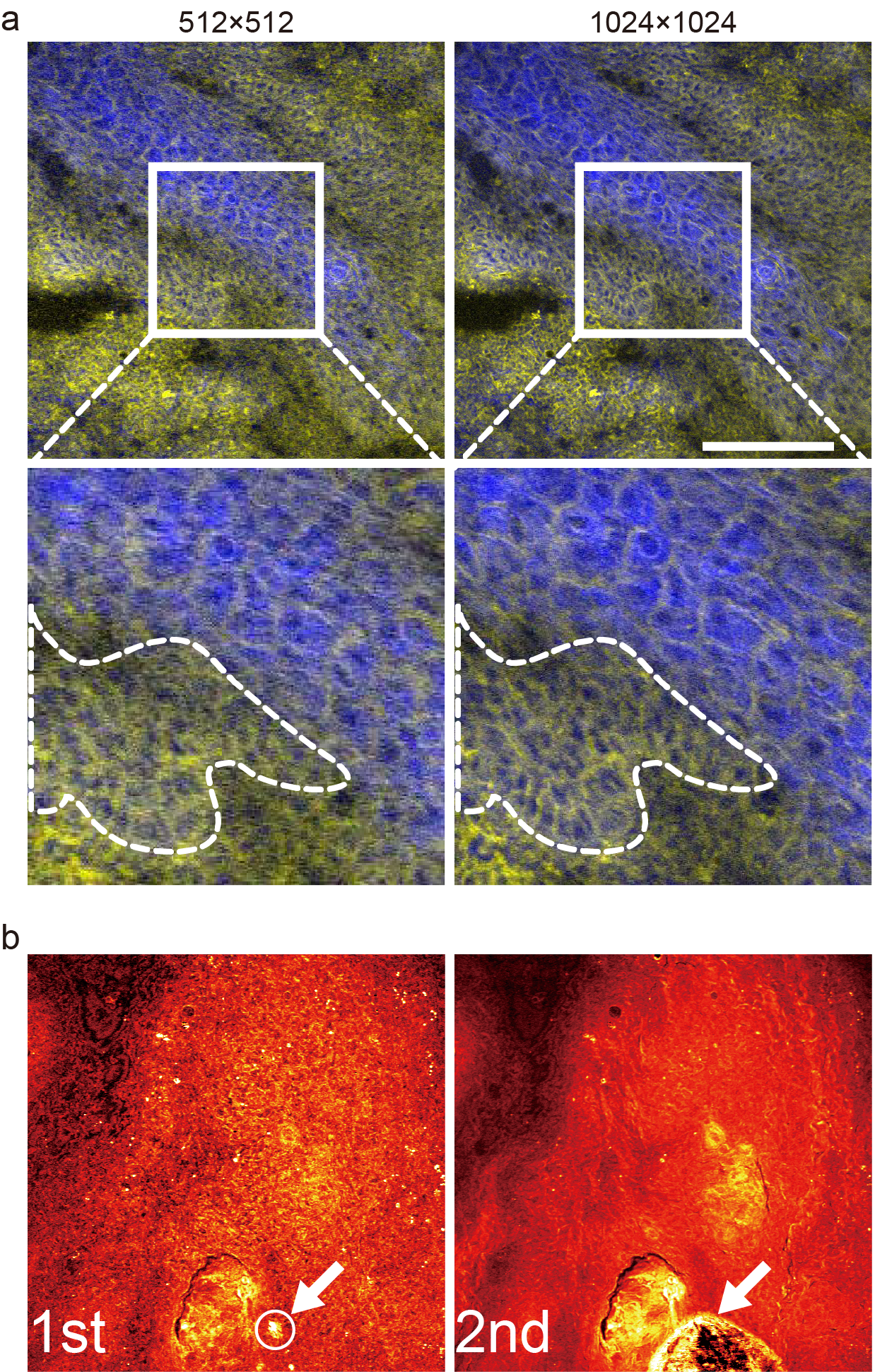

Supplement: S27 Fig — (TIF) [file pbio.3001699.s028.tif]

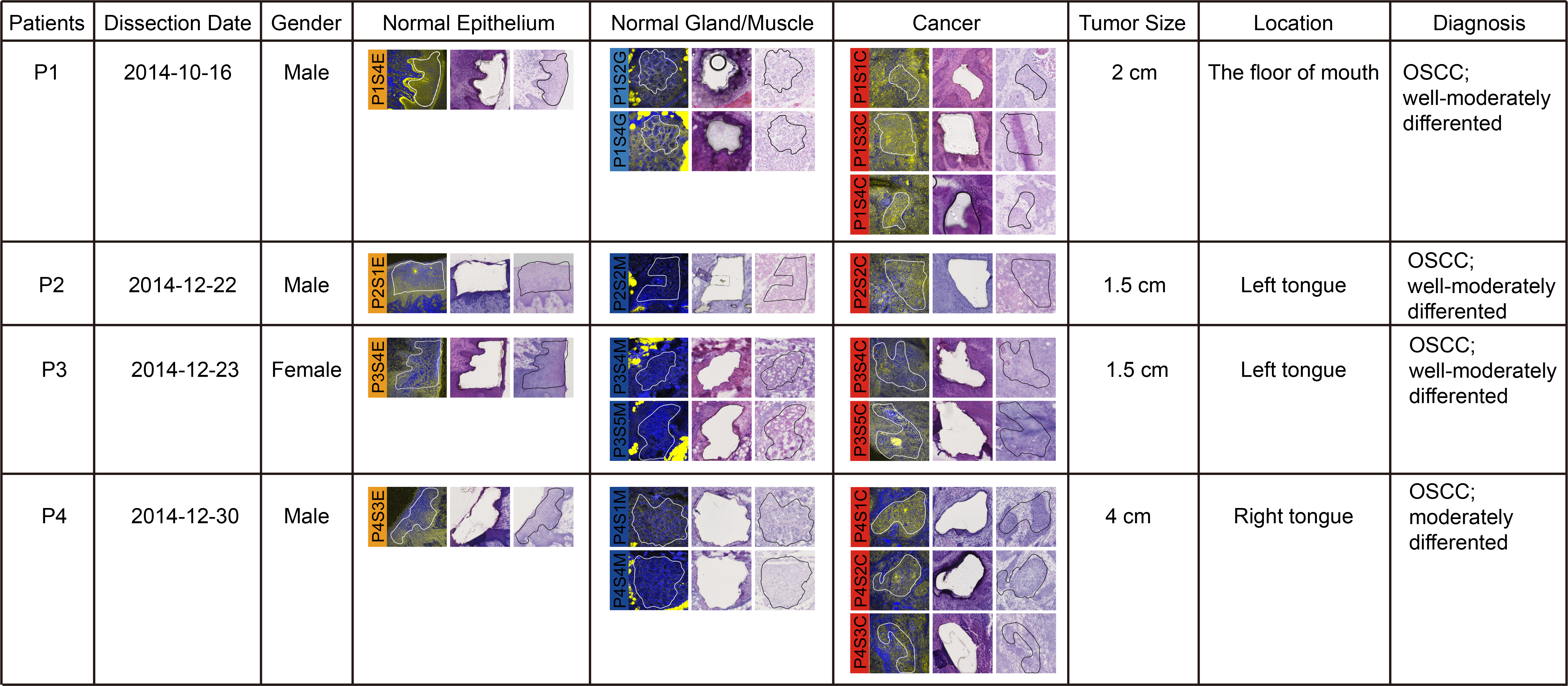

Supplement: S1 Table — (TIF) [file pbio.3001699.s029.tif]

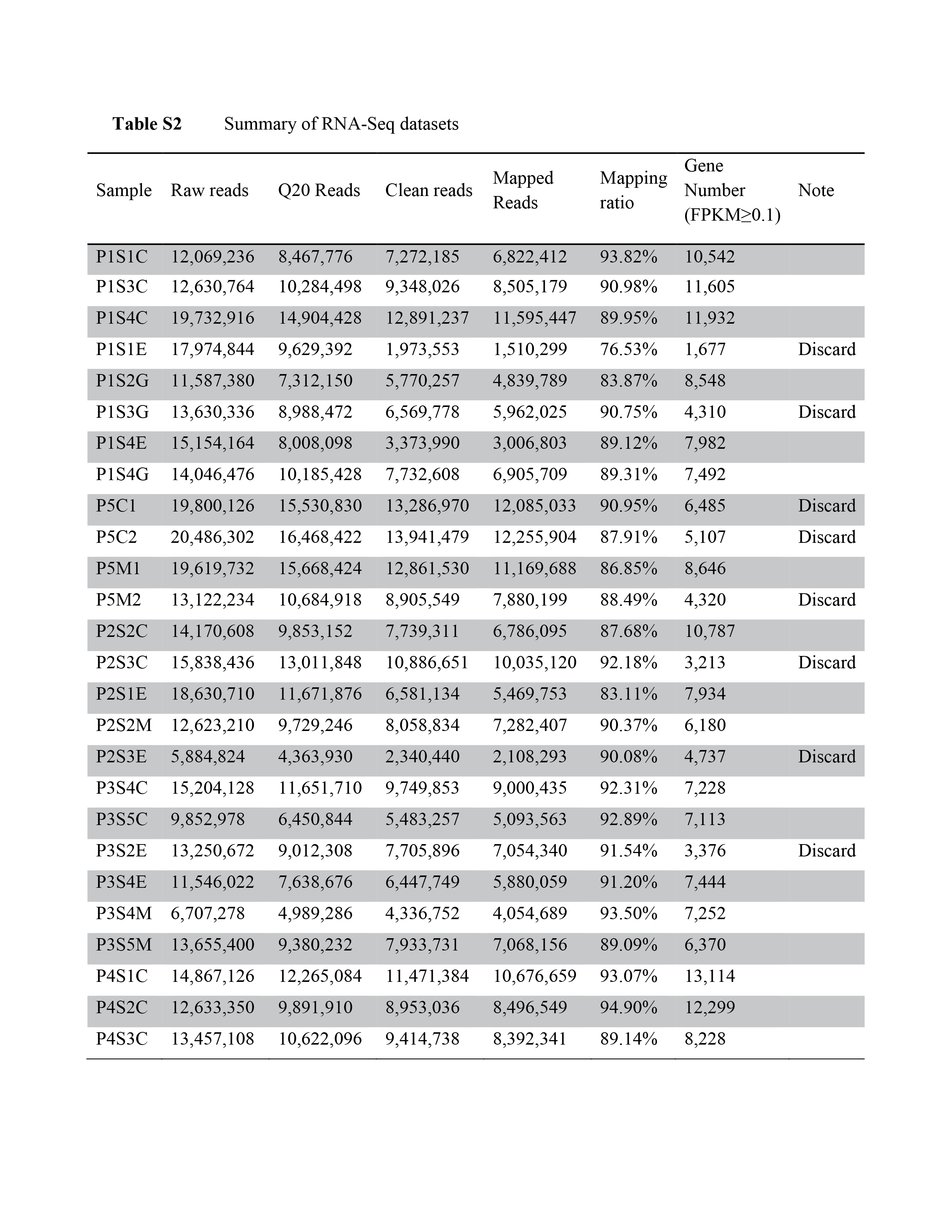

Supplement: S2 Table — (TIF) [file pbio.3001699.s030.tif]

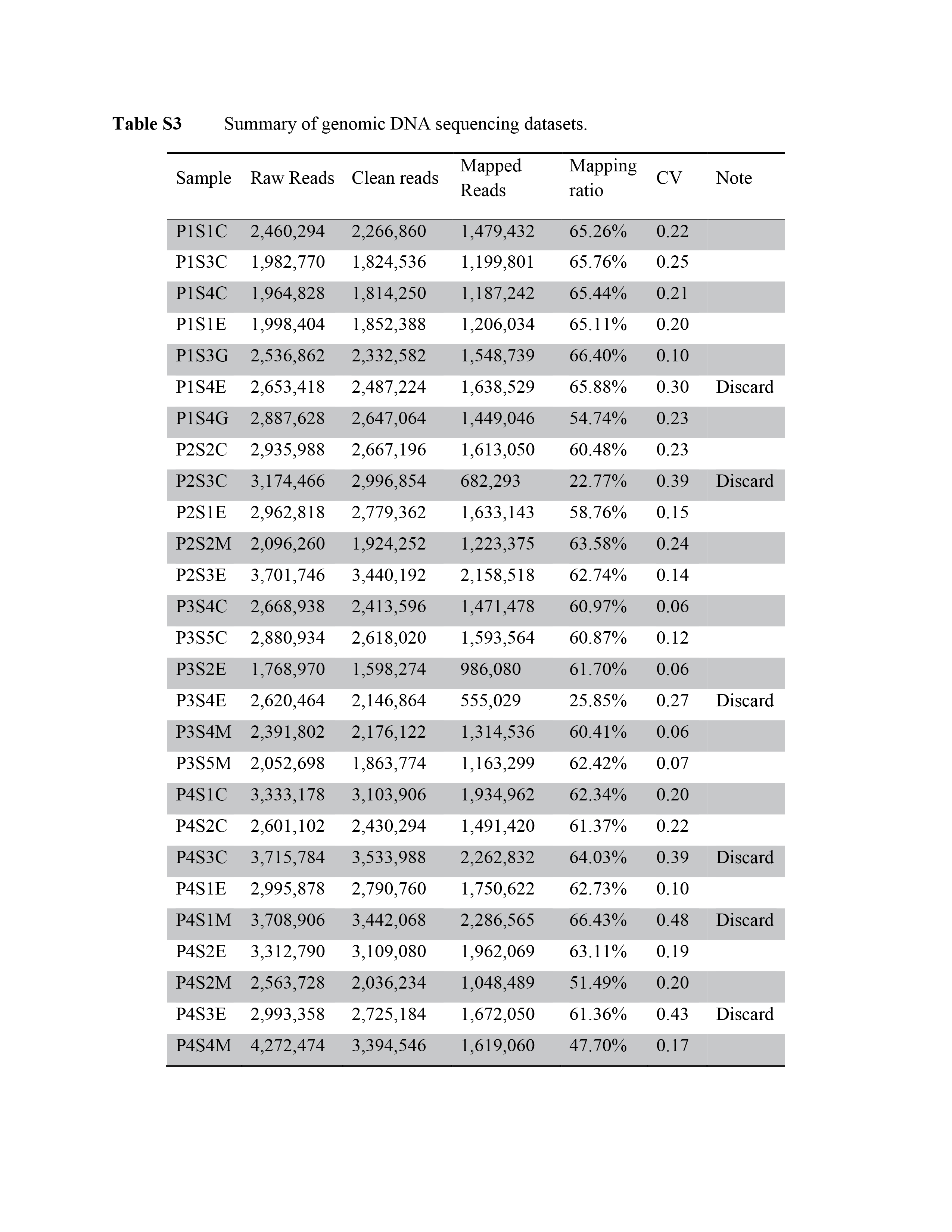

Supplement: S3 Table — (TIF) [file pbio.3001699.s031.tif]

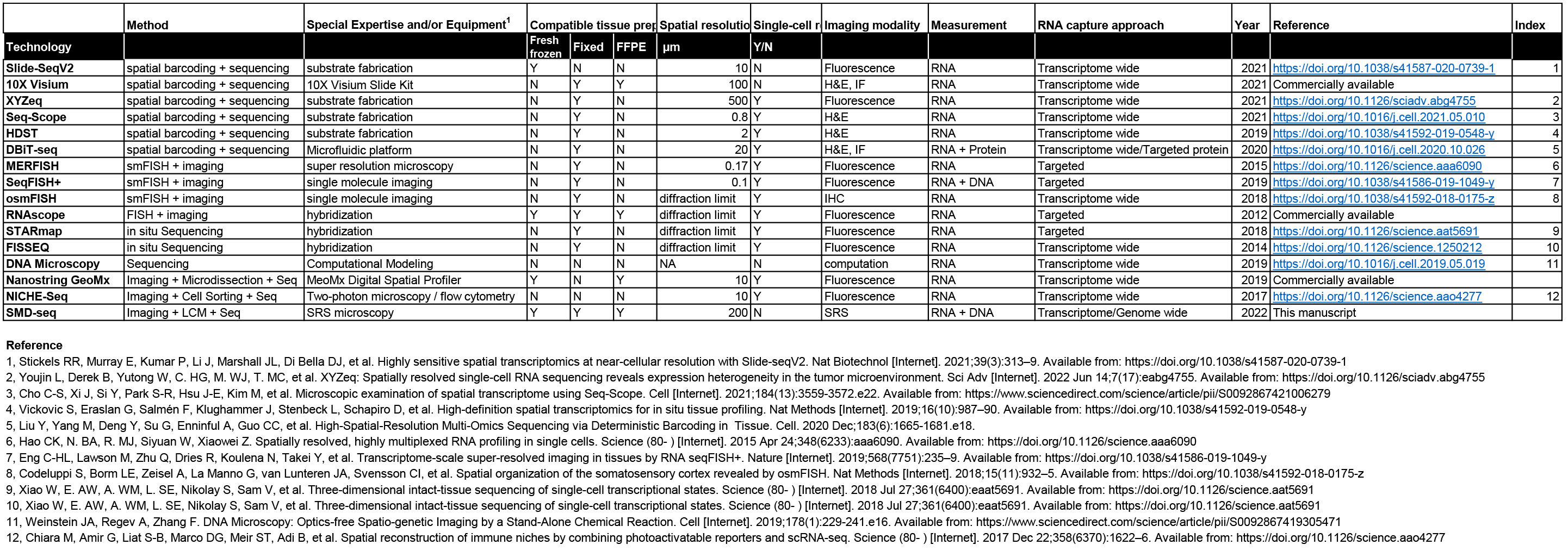

Supplement: S4 Table — (TIF) [file pbio.3001699.s032.tif]
